# Exploiting *Paraphaeosphaeria minitans* and Its Antifungal Metabolites as Bio‐Fungicides for Eco‐Friendly Management of Head Rot Disease in Cabbage

**DOI:** 10.1111/1751-7915.70309

**Published:** 2026-01-30

**Authors:** Meena V. Ruppavalli, Muthusamy Karthikeyan, Iruthayasamy Johnson, Sivaji Jeevanantham, Parthiban V. Kumaresan, Balakrishnan Prithiviraj, Sambasivam Periyannan

**Affiliations:** ^1^ Department of Plant Pathology Tamil Nadu Agricultural University Coimbatore Tamil Nadu India; ^2^ Centre for Crop Health University of Southern Queensland Toowoomba Queensland Australia; ^3^ Department of Plant Pathology Annamalai University Chidambaram Tamil Nadu India; ^4^ Department of Plant, Food and Environmental Sciences, Faculty of Agriculture Dalhousie University Truro Nova Scotia Canada; ^5^ School of Agriculture and Environmental Science University of Southern Queensland Toowoomba Queensland Australia

**Keywords:** field trial, molecular docking, molecular simulation, mycoparasitism, *Paraphaeosphaeria minitans*, *Sclerotinia sclerotiorum*

## Abstract

Cabbage head rot, caused by *Sclerotinia sclerotiorum*, threatens crop yield and quality. Among the 21 mycoparasitic fungi isolated from sclerotia, dormant structure and primary sources of inoculum for the pathogen, the strongest antagonism (78.51% mycelial growth inhibition) was observed in *Paraphaeosphaeria minitans* strain TNAU‐CM 1. Scanning electron microscopy (SEM) revealed its destructive colonisation, including pycnidia and pycnidiospore formation, with visible shrinkage and deformation of sclerotia. Gas chromatography–mass spectrometry (GC–MS) analysis identified 24 bioactive metabolites at the point of interactions between *P. minitans* TNAU‐CM 1 and *S. sclerotiorum* TNAU‐SS‐5 strains in dual‐culture assays. Further, crude metabolites from *P. minitans* TNAU‐CM 1 cultures inhibited the pathogen's mycelial growth by 54.4% at 100 ppm. In the molecular docking of 14 key compounds, linoleic acid and butyl octyl phthalate, well‐known antifungal compounds, displayed the highest binding affinity of −7.6 and −6.2 kcal/mol, respectively, against *Saccharomyces cerevisiae* cupin protein (1ZNP) YML079w, a homologue of SsYCP1, a YML079‐like cupin protein (YCP) and a virulence molecule from *S. sclerotiorum*. Field trials demonstrated that foliar application of *P. minitans* TNAU‐CM 1 stock solution (8–10 × 10^8^ spores per mL) at 5 mL/L dilutions significantly reduced disease incidence and the crops produced a yield of 41.37 tons/ha, comparable to chemical fungicide treatment (43.51 tons/ha). Thus, molecular interaction studies and field evaluations suggest that *P. minitans* TNAU‐CM 1 is a promising eco‐friendly alternative to synthetic fungicides for the management of cabbage head rot. Furthermore, our findings indicate that linoleic acid and butyl octyl phthalate are the key antifungal metabolites of *P. minitans*, active against *S. sclerotiorum* and will serve as potential candidates for developing bio‐fungicide formulations to control head rot in cabbage.

## Graphical abstract

Biocontrol of Cabbage Head Rot: *Paraphaeosphaeria minitans* produces antifungal metabolites, disrupts sclerotia of *Sclerotinia sclerotiorum*, and reduces disease incidence in cabbage under field conditions.

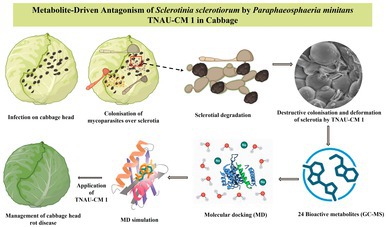

## 1 Introduction

Globally, India ranks as the second‐largest producer of vegetables, with 2.8% of the country's farmland devoted to their cultivation, accounting for 14% of global vegetable production. Cabbage ( *Brassica oleracea* var. *capitata* ) holds a prominent position in terms of area and production. In 2023–2024, the crop covered 423,000 ha, and with an average productivity of 23 metric tons/ha, the total production reached 9.8 million metric tonnes. The major cabbage‐producing states within India are West Bengal, Bihar, Odisha, Tamil Nadu and Maharashtra (Singh et al. 2023). Although cabbage cultivation has expanded in recent years in both India and worldwide, its production is adversely affected by the head rot disease caused by *Sclerotinia sclerotiorum* (Wang et al. 2024). In India, *S. sclerotiorum* incidences are reaching up to 49.07%, contributing to substantial yield reductions (Sivagnanapazham et al. 2022; Venkatesan et al. 2023). Global estimates indicate that *S. sclerotiorum* has the capability to cause yield losses of up to 90% in conducive environmental conditions. These reports emphasise the necessity for effective disease management strategies, although challenging due to the wide host range of *S. sclerotiorum*, with the ability to infect over 400 plant species, including cruciferous crops, legumes, and oilseeds (Danielewicz et al. 2025). Further, the pathogen spreads through infected plant debris, water, and mechanical transmission and can survive in soil for a long period as the sclerotia, the resting structure of *S. sclerotiorum*, remain dormant in soil for many years and germinate under cool, moist conditions, releasing airborne spores (Tejaswini et al. 2022).

Interestingly, mycoparasites such as *Trichoderma* spp., *Paraphaeosphaeria minitans* (previously referred to as *Coniothyrium minitans*), and *Gliocladium* spp. target sclerotia, thereby preventing the survival and spread of *S. sclerotiorum* (Wang et al. 2024). *Trichoderma* spp. are widely recognised for their rapid colonisation ability, production of antifungal metabolites, and broad‐spectrum antagonistic activity against soil‐borne pathogens, including *S. sclerotiorum* (Alqahtani 2025). Their mechanism of action typically involves mycoparasitism (direct hyphal penetration), competition for nutrients and space, and secretion of cell wall–degrading enzymes such as chitinases and glucanases. *Gliocladium* spp. (e.g., *G. roseum* ) suppress pathogen propagules primarily through competition and enzymatic degradation, producing antibiotic metabolites that inhibit hyphal growth. In contrast, *P. minitans* exhibits a highly specialised mode of parasitism, specifically targeting and degrading sclerotia of *S. sclerotiorum* through enzymatic breakdown of the melanised rind and medulla tissues, often leading to complete destruction of these survival structures in soil (Wang et al. 2025). This specificity is complemented by its ability to persist in the soil for extended periods and its proven ability to reduce the long‐term inoculum potential of *S. sclerotiorum* without significantly affecting non‐target microflora (Zhao et al. 2025). These ecological traits make it especially suited for sustainable, targeted biocontrol strategies compared to generalist mycoparasites.

In our study, mycoparasitic fungal isolates were obtained from sclerotia of *S. sclerotiorum* collected from naturally infected cabbage plants in the state of Tamil Nadu in India. As sclerotia are the primary survival and inoculum structures of the pathogen, this study was based on the ecological hypothesis that such field‐collected sclerotia harbour naturally occurring mycoparasites capable of suppressing *S. sclerotiorum*. This premise formed the basis for screening and identifying the most effective antagonist for potential biocontrol applications. Additionally, mycoparasites were known to invade and colonise sclerotia through secretion of enzymes and secondary metabolites, including terpenes, alcohols, ketones, aldehydes, esters, sulphur and nitrogen‐containing compounds. Several of these metabolites, including linoleic acid and phthalate derivatives, have been reported in earlier microbial antifungal studies; however, their specific activity against *S. sclerotiorum* virulence proteins under field conditions remains underexplored. These antimicrobial compounds were found to target SsYCP1, a YML079‐like cupin protein (YCP) that plays a crucial role in the pathogenicity of *S. sclerotiorum* (Fan et al. 2021). The SsYCP1 is highly expressed and secreted during infection, and its ectopic overexpression significantly enhances virulence. Notably, SsYCP1 is the first reported YCP with a secretory function, suggesting its potential role as an effector (Singh et al. 2024). Interestingly, YCP in other ascomycetes lacks a secretion signal peptide, indicating that SsYCP1 may have evolved as a unique secretory protein to facilitate *S. sclerotiorum* infection (Fan et al. 2021). Based on these unique structural and functional attributes, SsYCP1 is considered a potential therapeutic target, as disrupting or inhibiting this effector‐like protein could directly impair the pathogen's virulence without necessarily killing it. As no crystal structure of SsYCP1 is available, the homologous *Saccharomyces cerevisiae* cupin protein (1ZNP) was used as a structural reference for homology modelling and docking studies. This aligns with mechanism‐driven biocontrol strategies, wherein microbial metabolites or enzymes specifically disarm pathogen virulence factors, thereby reducing disease severity while preserving beneficial microflora. While prior studies have characterised SsYCP1 and identified inhibitory metabolites in vitro, there remains a need to integrate computational prediction of metabolite–target interactions with practical, field‐level validation for disease management.

Molecular docking predicts the binding conformation and affinity of small molecules (ligands) within the active site of target proteins, providing valuable insights into biomolecular interactions. Meanwhile, molecular dynamics (MD) simulation offers a dynamic perspective by modelling the structural flexibility, stability, and conformational changes of biomolecules over time (Childers and Daggett 2017). These techniques have become essential tools in drug discovery and structural biology, facilitating the identification of potential therapeutic targets. In this context, the present study integrates laboratory assays, Gas chromatography–mass spectrometry (GC–MS) metabolite profiling, *in silico* interaction analysis, and field trials to assess the practical biocontrol potential of *P. minitans* against cabbage head rot. Therefore, our current study was undertaken to: (i) analyse the diversity of mycoparasitic fungi associated with the sclerotia of *S. sclerotiorum*, the causal agent of cabbage head rot; (ii) assess the antifungal organic compounds produced during their interaction; (iii) predict the inhibitory activity and target proteins using molecular docking and simulation dynamics; (iv) evaluate the efficacy of a liquid formulation under both glasshouse and field conditions.

## 2 Experimental Procedures

### 2.1 *Sclerotinia sclerotiorum* Strain and the Inoculation Method

The highly virulent *S. sclerotiorum* strain TNAU‐SS‐5 (National Centre for Biotechnology Information [NCBI] accession number MZ379266.1), maintained at the Department of Plant Pathology, Tamil Nadu Agricultural University (TNAU), Coimbatore, Tamil Nadu, India, was used for this study (Ruppavalli et al. 2022; Venkatesan et al. 2023). For inoculation, a 9 mm culture disc of the strain was placed on a pinpricked head portion of the cabbage plant, then wrapped with parafilm, and the entire plant was enclosed in a transparent polyethylene bag to maintain humidity while allowing light penetration. Pinpricked healthy cabbage plants without fungal discs served as the negative control. Both inoculated and uninoculated cabbage plants were maintained at 20°C ± 2°C for 7 days and observed at regular intervals for symptom development. This inoculation assay was performed to confirm the pathogenicity and virulence of the strain, establishing a reliable disease model for subsequent evaluation of biocontrol agents.

### 2.2 Isolation and Characterisation of Mycoparasites From Sclerotia

Sclerotial bodies were collected from *S. sclerotiorum*‐infected cabbage fields at Nanjanadu village (11°24′56.4″ N, 76°42′23.1″ E), Nilgiris district in Tamil Nadu state of India, where the incidence of head rot was observed recently (Ruppavalli et al. 2022). Sclerotia collected from the fields were used instead of artificially generated sclerotia to represent natural infection and capture mycoparasitic associations in the field environment. To ensure systematic and unbiased sampling, a grid‐based technique was used across a 500‐square‐meter field, allowing uniform coverage and representative data collection. A total of 100 sclerotial bodies were gathered from plants showing varying levels of infection, categorised as low, moderate, and high based on visible symptoms like lesion size and plant rot (Rahman et al. 2020). To isolate mycoparasitic fungi, the collected sclerotial bodies were first surface‐sterilised with 1% sodium hypochlorite solution for 3 min and then rinsed twice with sterile water. Three sclerotia were placed equidistantly in sterilised polystyrene Petri dishes (100 × 15 mm) containing 50% strength Potato Dextrose Agar (PDA) medium, sealed with parafilm, and incubated at 20°C ± 2°C for 15 to 20 days (Tomprefa et al. 2011). Sclerotia that developed pigmented hyphal growth, ranging from yellowish white to dark brown, were presumed to be colonised by mycoparasitic fungi. The fungal mycelium from these sclerotia was aseptically transferred to fresh Petri dishes containing full‐strength PDA medium (Campbell 1947). Key morphological features, including colony morphology, colour, size, hyphal and growth characteristics, were observed using an Olympus BX9‐CBH clinical microscope. Fungal isolates were purified using the single‐spore isolation method (Zeng et al. 2012).

### 2.3 Molecular Characterisation of the Mycoparasites

Genomic DNA from the mycoparasitic fungal isolates was extracted using the GeneJET Plant Genomic DNA Purification Mini Kit (Thermo Fisher Scientific, catalogue numbers K0791 and K0792), following the manufacturer's protocol. Amplification of the internal transcribed spacer (ITS) region was performed using forward (5′‐TCCGATGGTGAACCTGCGG‐3′) and reverse (5′‐TCCTCCGCTTATTGATATGC‐3′) primers. The PCR amplification was carried out with an initial denaturation at 95°C for 10 min, followed by 35 cycles of denaturation at 94°C for 1 min, annealing at 58°C for 1 min, and extension at 72°C for 1 min. A final extension was performed at 72°C for 10 min. The PCR products were examined using 1% agarose gel electrophoresis to confirm the amplification of a ~580 bp fragment (Redecker et al. 1997). The amplified products were subsequently purified using QIAquick PCR Purification Kit (catalogue numbers 28104 and 28106), and Sanger dideoxy sequencing was conducted at Chromos Biotech Pvt. Ltd., Bangalore, India. The sequences were submitted to NCBI, and their similarities were assessed using BLASTN version 2.0. Confirmation of *P. minitans* was done using rRNA sequence‐specific primers Cm spp. 1F (5′ CCCCAGGTGGTAAGGTGAAA‐3′) and Cm spp. 1R (5′ TACTAGATGCAAAAAAGGTTTATCAG‐3′) as described in Sivagnanapazham et al. (2022). Nucleotide sequences from Sanger sequencing were aligned with each other using the ClustalW algorithm. Phylogenetic relationships were then reconstructed in MEGA version 11 using the Maximum Likelihood method with the Tamura–Nei substitution model. To assess the reliability of the inferred relationships, bootstrap analysis was performed with 1000 replicates (Jeevanantham et al. 2025).

### 2.4 Antifungal Activity of the Mycoparasites Against *S. sclerotiorum*

The efficacy of mycoparasitic fungal isolates obtained from sclerotia was evaluated against the virulent *S. sclerotiorum* TNAU‐SS‐5 strain using a dual‐culture technique. A 9 mm diameter mycelial disc of each 20‐day‐old parasitic fungal isolate was placed on one side of a PDA Petri dish at a distance of 1 cm from the periphery. Similarly, a 9 mm diameter mycelial disc from a 7‐day‐old TNAU‐SS‐5 culture was placed on the opposite side of the Petri dish. The inoculated dishes were incubated at 20°C ± 2°C under 12‐h fluorescent light conditions for 14 days (Wang et al. 2024). During the incubation, the plates were monitored for the development of inhibition zones between the growth of the mycoparasites and *S. sclerotiorum* TNAU‐SS‐5. Each fungal isolate was tested in triplicate, with 10 plates maintained per replication (Bitsadze et al. 2015). The percentage reduction in mycelial growth was calculated relative to the untreated control using the appropriate formula,

C−T/C×100

where *C* denotes the mycelial growth of the pathogen in control and *T* denotes the mycelial growth of the pathogen in the dual culture technique.

### 2.5 Structural Characterisation of *P. minitans* TNAU‐CM 1 Using Scanning Electron Microscopy (SEM)

To investigate the colonisation behaviour of *P. minitans* TNAU‐CM 1, pathogenic sclerotial bodies were inoculated with a spore suspension (1 × 10^6^ spores/mL) by spray application of 5 mL onto the surface. After 30 days post‐inoculation (DPI), the infected sclerotia, which had been fully parasitised, were carefully bisected using a razor blade. To provide detailed insights into the interaction between the mycoparasite and the sclerotial tissue, parasitised sclerotial halves were then mounted onto standard copper SEM stubs using dual‐bonded sticky tape. To minimise damage to the samples and enhance structural contrast, the specimens were sputter‐coated with a thin conductive layer of gold alloy (300 nm thickness) using an EMITECH ion sputter coater. The samples were then examined under a Field Emission SEM (FE‐SEM), model FAI QUANTA 250, at an accelerating voltage of 15 kV to capture high‐resolution images of the colonisation process.

### 2.6 | Preparation of Crude Enzyme Extract of *P. minitans* TNAU‐CM 1

Potato Dextrose Broth (PDB) was utilised to evaluate the production of defence‐related enzymes by substituting the standard carbon source with various alternatives, including sucrose, xylan, carboxymethyl cellulose (CMC), laminarin, and chitin. The concentrations of sucrose, xylan, and CMC were maintained at 1% (w/v), while laminarin and chitin were incorporated at 0.5% and 0.2%, respectively. The pH of the media was adjusted to 6.0 using 0.1 N NaOH, followed by autoclaving at 121°C for 20 min. Each flask was inoculated with 9 mm mycelial discs derived from actively growing cultures of the highly effective mycoparasitic fungi, *P. minitans* TNAU‐CM 1. The inoculated flasks were incubated on a rotary shaker at 180 rpm under controlled conditions of 20°C ± 2°C. Samples for enzyme activity assays were collected at 7, 15, and 20 DPI to assess the impact of different carbon sources on enzyme production (Guigón‐López et al. 2015). The enzyme assay was conducted using *P. minitans* TNAU‐CM 1 inoculated in media containing different carbon sources, which were covalently labelled with Remazol Brilliant Blue (RBB). The substrates included fungi‐cellulose‐RBB, fungi‐xylan‐RBB, fungi‐β‐1,3‐glucan‐RBB, and fungi‐chitin‐RBB, corresponding to the assessment of endocellulase, endoxylanase, β‐1,3‐glucanase, and endochitinase activities, respectively. The RBB‐labelled substrate assay specifically detects endo‐type glycoside hydrolase activity, as internal cleavage of the polymer backbone releases soluble dye‐linked fragments, while exo‐acting enzymes attacking terminal residues were inefficient in solubilising the dye (Guigón‐López et al. 2015). The assay protocol involved mixing 100 μL of the dye‐labelled substrate with 100 μL of 0.1 M sodium acetate buffer (pH 5.0) and 100 μL of enzyme extract collected at different time intervals (7, 15, and 20 DPI). A control reaction containing 100 μL of buffer without an enzyme extract served as the blank. The reaction mixtures were incubated in a water bath at 40°C for 1 h. Following incubation, 100 μL of 1 N HCl was added to terminate the reaction, and the tubes were placed on ice for 10 min. The samples were then centrifuged at 2500 rpm for 10 min, and 175 μL of the resulting supernatant was carefully transferred to 96‐well microtiter plates. The optical density was measured at 595 nm using an ELISA reader to quantify enzyme activity.

### 2.7 Determining the Antifungal Activity of *P. minitans* TNAU‐CM 1 Using Gas Chromatography–Mass Spectrometry (GC–MS)

The crude culture filtrate produced by *P. minitans* TNAU‐CM 1 was analysed using GC–MS to identify its bioactive compounds (BCs). To specifically analyse metabolites produced during interaction with *S. sclerotiorum* TNAU‐SS‐5, the BCs were isolated from the inhibition zone formed in dual culture assays on PDA medium, following the method of Cawoy et al. (2015) with slight modifications. The inhibition zones were carefully excised from the agar using a sterile scalpel, yielding approximately 150 mg of agar containing BCs. This excised material was mixed with 500 μL of HPLC‐grade acetonitrile and water (1:1, v/v) and sonicated twice for 30 s at 30% power to enhance compound extraction. The resulting mixture was homogenised, vortexed, and centrifuged to remove particulate matter. The supernatant was concentrated to 150 mL by evaporation under reduced pressure using a vacuum flask evaporator, and any remaining solvent was air‐dried in sterile Petri plates (Ravi et al. 2022). The dried metabolites were subsequently re‐dissolved in HPLC‐grade methanol for further analysis. To distinguish metabolites produced specifically by the mycoparasite, the pathogen, or their interaction, comparative samples were prepared from (i) PDA medium alone (control), (ii) PDA inoculated solely with *P. minitans* TNAU‐CM 1, and (iii) PDA inoculated solely with *S. sclerotiorum* TNAU‐SS‐5. These controls were processed identically to the dual‐culture samples, allowing for the identification of unique metabolites associated with the interaction. Prior to GC–MS analysis, the extracts were filtered through a 0.25 μm polyvinylidene difluoride (PVDF) syringe filter to remove any residual impurities. Additionally, a sodium sulphate cartridge was used to eliminate moisture from the samples, which were then concentrated to 1 mL using a TurboVap evaporator at 55°C under a nitrogen stream. The samples prepared were subjected to GC–MS analysis using a Clarus SQ 8C GC–MS system (Perkin Elmer, Akron, OH, USA). The identification of BCs was performed by comparing the mass spectra with reference data from the National Institute of Standards and Technology (NIST) library (NIST14 software), allowing accurate characterisation of the metabolites produced by highly effective mycoparasite.

### 2.8 Detection of Antifungal Activity of Crude Metabolite From *P. minitans* TNAU‐CM 1 Using Agar Well Diffusion Approach

For the antifungal assay, sterile Petri dishes containing 15 mL of solidified PDA were taken and the agar wells were created with a diameter of 9 mm using a sterile cork borer, positioned symmetrically 1 cm from the periphery of the plate. A 9 mm actively growing mycelial disc of *S. sclerotiorum* TNAU‐SS‐5 strain was placed at the center of each plate. The crude culture filtrate used in this assay was obtained exclusively from the mycoparasite grown in pure culture, without the presence of *S. sclerotiorum*, to ensure that the metabolites analysed were solely produced by the mycoparasite and not by the pathogen. This distinction was made to address the possibility of mixed metabolite profiles arising from inhibition assays. Crude culture filtrate was prepared following the methods described by Kim et al. (2022) with slight modifications. A 9 mm mycelial disc of the highly effective mycoparasite *P. minitans* TNAU‐CM 1 was inoculated into a 500 mL conical flask containing 250 mL of PDB. After 15 DAI, the resulting mycelial mat was harvested, freeze‐dried, and ground into a fine powder using a sterile mortar and pestle. The powdered samples were stored at −80°C for subsequent crude metabolite extraction. For extraction, 100 mg of dried powder was mixed with 1 mL of 100% methanol (MeOH) containing 10 μL of 2‐chloro‐L‐phenylalanine (1 mg/mL) as an internal standard. The mixture was homogenised using an MM400 mixer mill at a frequency of 45 s^−1^ for 10 min, followed by sonication for 5 min. The extracts were then centrifuged at 13,500 rpm for 15 min at 4°C. The resulting supernatant was filtered through a 0.22 μm polytetrafluoroethylene (PTFE) syringe filter and subsequently dried using a speed‐vacuum concentrator. The dried extract was reconstituted in 100% methanol to achieve a final concentration of 100 ppm for use in antifungal activity assays against *S. sclerotiorum* TNAU‐SS‐5 strain. Crude extracts of a highly effective mycoparasite were added to the wells at concentrations of 25, 50, 75, and 100 ppm, with 10 μL dispensed per well. Plates were incubated at 20°C ± 2°C for 3 days. Each concentration was tested in triplicate, and HPLC‐grade methanol served as the negative control. The percentage inhibition of mycelial growth relative to the control was calculated to assess the antifungal efficacy of the crude metabolites. Percent inhibition of mycelial growth over control was calculated using the formula in Section 2.4.

### 2.9 Virtual Screening of Active Metabolites Through Molecular Docking

To predict the inhibitory potential of the antifungal compounds, molecular docking interaction and binding studies were conducted using AutoDock Vina 1.1.2 and AutoDock Tools (Eberhardt et al. 2021; Fan et al. 2021). The SsYCP1 protein sequence (SS1G_06230) was retrieved from NCBI, and the crystal structure of the homologous *S. cerevisiae* cupin protein YML079w (PDB ID: 1ZNP) was obtained from the Protein Data Bank, and the protein sequence was retrieved. Pairwise sequence similarity was assessed using BLASTp, and conservation of the β‐barrel cupin domain was analysed through multiple alignment with COBALT. A 3D homology model of SsYCP1 was generated in SWISS‐MODEL using 1ZNP as a template. Structural alignment and stereochemical validation were performed using PROCHECK Ramachandran plots. Before docking, all water molecules were removed using AutoDock Tools, and polar hydrogen atoms were added along with Colman charges. The processed file was then saved in PDBQT format for further analysis. The docking grid box was configured with X, Y, and Z dimensions set to 60 Å and centred on key residues of the protein. Ligand structures were retrieved from PubChem in 3D format. For compounds available only in 2D, ChemSketch software was utilised to construct their 3D molecular structures, which were then converted appropriately (Li et al. 2004). The lowest energy conformations were selected for further analysis. For comparison, NATIVO, a commercial dimethylase inhibitor (DMI) composed of Tebuconazole (50%) and Trifloxystrobin (25%), was also docked (Molaei et al. 2020), and its binding energy was evaluated alongside the other compounds. Docking simulations were performed for all compounds identified through GC–MS analysis, enabling evaluation of their binding affinity with the target protein. Among them, the highest‐ranked compounds, based on binding energy, were further analysed to identify their potential binding sites. To better understand the molecular interactions, BIOVIA Discovery Studio was employed to generate 2D interaction diagrams, providing a detailed visualisation of compounds' interaction with the pathogenic protein at the molecular level. The docking study evaluated key parameters, including binding affinity (kcal/mol), the number of amino acid residues involved in hydrogen bonding, and the specific amino acids contributing to these interactions. The biomolecule with higher binding affinity was purchased from Sigma‐Aldrich and tested against *S. sclerotiorum* TNAU‐SS‐5 strain using the agar well diffusion method. Four concentrations (250, 500, 750, and 1000 ppm) were prepared from a 10,000 ppm stock. PDA plates were poured, and 7 mm wells were filled with each concentration. A 9 mm mycelial disc from a 7‐day‐old culture was placed on the agar, and plates were incubated at 25°C ± 2°C for 7 days. Zones of inhibition were measured, and percent mycelial growth reduction was calculated relative to the control. Although confirmatory analyses such as LC–MS/MS or direct GC–MS analysis of authentic standards were not performed for metabolite identification, two major GC–MS‐detected compounds were subsequently procured as pure analytical standards and individually evaluated for their inhibitory activity in vitro.

### 2.10 Molecular Dynamics (MD) Simulations

The MD simulations of the top‐ranked protein‐ligand complexes were performed using CABS‐flex V 2.0 (http://biocomp.chem.uw.edu.pl/CABSflex2) and the iMODS server (http://imods.chaconlab.org) to evaluate protein flexibility and stability (Kuriata et al. 2018; Shanmugam and Jeon 2017). The dynamic behaviour of the 1ZNP protein was examined by analysing backbone flexibility, variations in binding interactions, and possible conformational shifts during the simulation. CABS‐flex was utilised to estimate root‐mean‐square fluctuation (RMSF) values, which reflect changes in protein mobility upon ligand binding and indicate the conformational changes within the protein–ligand complex. Simulations were performed for 10 ns, using default settings for all other parameters. To further evaluate the stability and molecular behaviour of the docked protein‐ligand complexes, MD simulations were performed using the iMODS server. This platform was utilised to analyse the structural dynamics and mobility of the docking complexes. The iMODS server provided an analysis of protein‐ligand interaction stability using various parameters, including deformability, B‐factor, eigenvalues, variance, covariance mapping, and elastic network analysis. The docked PDB files were uploaded to the iMODS server with default settings applied (López‐Blanco et al. 2014). Deformability and B‐factor analyses help to understand the mobility profiles of docked proteins, with deformability peaks indicating regions of high flexibility. Additionally, B‐factor plots were used to compare normal mode analysis (NMA) results with the PDB field of the complexes, providing further insights into their structural stability (Shadidizaji et al. 2024).

### 2.11 Development of Liquid Formulation of *P. minitans* TNAU‐CM 1 Against *S. Sclerotiorum* TNAU‐SS‐5

#### 2.11.1 Optimisation and Evaluation of Bioformulations

To optimise a suitable synthetic broth for developing a liquid formulation of *P. minitans* TNAU‐CM 1, four different liquid media were evaluated viz., malt yeast broth, nutrient yeast sucrose broth, molasses yeast broth, and jaggery yeast broth (Yasin et al. 2020). Each broth was prepared and sterilised by autoclaving at 121°C for 30 min to ensure aseptic conditions. Subsequently, a 9 mm mycelial disc from an actively growing culture of TNAU‐CM 1 was inoculated into 500 mL conical flasks, each containing 250 mL of one of the four sterilised broths. The inoculated flasks were incubated at 20°C ± 2°C for 15 days under static conditions. After the incubation period, the dry mycelial biomass (expressed in grams) and spore concentration (number of spores per mL) of effective mycoparasite were quantified for each broth to determine the most suitable medium for liquid formulation development. Similarly the prevalent biocontrol agents viz., *Trichoderma asperellum* TRI 15 (KX533985) and the bacterial antagonist *Bacillus subtilis* Bby 57 (MG241251) were obtained from the Culture Collection Centre, Department of Plant Pathology, TNAU, Coimbatore, India (Sreenayana et al. 2022; Vinodkumar et al. 2017). These strains were chosen as reference microbial agents because they represent well‐established and commercially available biocontrol options widely adopted in vegetable disease management in Tamil Nadu, thereby serving as benchmark standards for comparison with *P. minitans* TNAU‐CM 1. These agents, along with the *P. minitans* TNAU‐CM 1, were screened for their antagonistic activity against *S. sclerotiorum* TNAU‐SS‐5, with the standard fungicide Tebuconazole + Trifloxystrobin (commercially known as NATIVO) serving as the chemical control (Molaei et al. 2020). Although an *in silico* docking study was conducted to examine the binding of two molecules with the protein 1ZNP, these molecules were not used in spray treatments because the docking analysis served only as a preliminary computational screening. The in vivo experiments focused exclusively on formulated biocontrol agents and standard fungicides.

#### 2.11.2 Mass Multiplication of *S. sclerotiorum*

The pathogen, *S. sclerotiorum* (strain TNAU‐SS‐5) was mass‐cultured using a sand‐maize medium following the protocol of Whipps et al. (2008). The medium was prepared by mixing sand and finely ground maize seeds in a 19:1 ratio, moistened with 100 mL of water per 500 g of the mixture, and packed into polypropylene bags. The mixture was sterilised by autoclaving twice on alternate days. After cooling, it was inoculated with 9 mm mycelial discs of *S. sclerotiorum* (TNAU‐SS‐5) and incubated at 25°C ± 2°C for 15 days to promote fungal growth. Following incubation, the colonised medium was pooled, air‐dried, and ground into a fine powder to serve as inoculum. For the glasshouse trials, a sterilised potting mixture composed of red soil, sand, and farmyard manure (FYM) in a 1:1:1 ratio (w/w/w) was prepared, sterilised twice, and filled into 30 cm diameter earthen pots. Each pot was inoculated with 10 g of the powdered *S. sclerotiorum* inoculum to establish the pathogen for experimental studies.

#### 2.11.3 Efficacy of Bioformulation Against Cabbage Head Rot Under Glass House Conditions

The study was designed following a completely randomised block design (CRBD) to evaluate the efficacy of biological control agents and a fungicide against *S. sclerotiorum* (TNAU‐SS‐5) in cabbage under controlled glasshouse conditions. The greenhouse trials evaluated only formulated biocontrol agents and the fungicide, as the docking study served solely as preliminary computational screening. The stock of the biocontrol agent used consist of 8–10 × 10^8^ spores (for *P. minitans* and *T. asperellum*)/colony‐forming units ( *B. subtilis* ) per mL. Six treatment (T) groups were established: T1—Foliar spray (FS) of *P. minitans* TNAU‐CM 1 stock solution at 5 mL/L, T2—FS of *T. asperellum* (TRI 15) stock solution at 5 mL/L, T3—FS of *B. subtilis* (Bbv 57) stock solution at 5 mL/L, T4—FS of a fungicide combination (Tebuconazole + Trifloxystrobin) at 1.5 g/L, T5—pathogen‐inoculated control, and T6—healthy, uninoculated control. Foliar applications were administered at three intervals: 30, 45, and 60 days after transplanting (DAT). Cabbage seedlings were transplanted into pots filled with a sterilised substrate comprising red soil, sand, and farmyard manure (1:1:1, w/w/w), pre‐inoculated with *S. sclerotiorum* (TNAU‐SS‐5) for pathogen challenge. Each treatment was replicated thrice, with 10 plants per replication. A water‐treated control was maintained to serve as an additional baseline reference. Plants were grown under controlled glasshouse conditions, with temperatures set at 22°C during the day and 20°C at night, and a photoperiod of 16 h of light and 8 h of darkness (Yasin et al. 2020). Disease incidence was monitored and recorded at 30, 45, and 60 DAT to determine the comparative effectiveness of each treatment using the appropriate formula,

$$\begin{array}{c} \mathrm{Percent}\;\text{disease incidence}=\mathrm{No}.\;\text{of infected plants}/\\ \;\text{Total}\;\mathrm{no}.\;\text{of plants}\times 100 \end{array}$$

### 2.12 Induction of Defence Related Enzymes

Cabbage leaves were treated with the specified biocontrol agents and subsequently challenge‐inoculated with *S. sclerotiorum* (TNAU‐SS‐5). Leaf samples were harvested at 0 h, and on the 3rd, 5th, 7th, and 9th DPI, then stored at 25°C for subsequent analyses.

#### 2.12.1 Peroxidase Activity

Peroxidase activity was followed by the methods of Abbattista Gentile et al. (1988), with slight modifications. One g of fresh leaf tissue was homogenised in a pre‐chilled mortar and pestle using 1 mL of 0.1 M phosphate buffer (pH 7.0). The homogenate was centrifuged at 10,000 rpm for 20 min at 4°C, and the resulting supernatant was collected as an enzyme extract. The reaction mixture consisted of 1.5 mL of 0.05 M pyrogallol, 0.1 mL of the enzyme extract, and 0.5 mL of 1% hydrogen peroxide. The change in absorbance was measured at 420 nm using a UV–visible spectrophotometer at 30‐s intervals for a total of 3 min, starting immediately after incubation (0 s). Peroxidase activity was expressed as the change in absorbance per minute per gram of fresh tissue (ΔA_420_ min^−1^ g^−1^ FW).

#### 2.12.2 Assay of Polyphenol Oxidase (PPO) Activity

Polyphenol oxidase (PPO) activity was determined following the method of Srivastava (1987) with slight modifications. One g of fresh leaf tissue was homogenised in a pre‐chilled mortar and pestle using 2 mL of 0.1 M sodium phosphate buffer (pH 6.5). The homogenate was centrifuged at 20,000 rpm for 15 min at 4°C, and the resulting supernatant was collected as the enzyme extract. The reaction mixture consisted of 1.5 mL of 0.1 M sodium phosphate buffer (pH 6.5) and 200 μL of the enzyme extract. The reaction was initiated by adding 200 μL of 0.01 M catechol. The change in absorbance was measured at 490 nm using a UV–visible spectrophotometer at 30‐s intervals for 3 min, starting immediately after incubation (0 s). PPO activity was expressed as the change in absorbance per minute per gram of fresh tissue (ΔA_490_ min^−1^ g^−1^ FW).

#### 2.12.3 Assay of Phenylalanine Ammonia‐Lyase (PAL) Activity

The PAL activity was assessed following the method of Dickerson et al. (1984) with slight modifications. One g of fresh leaf tissue was homogenised in a pre‐chilled mortar and pestle using 3 mL of ice‐cold 0.1 M sodium borate buffer (pH 6.5) containing 1.4 mM 2‐mercaptoethanol and 50 mg of insoluble polyvinylpyrrolidone (PVP). The homogenate was centrifuged at 20,000 rpm for 15 min at 4°C, and the resulting supernatant was collected as the enzyme extract. For the PAL activity assay, 0.4 mL of the enzyme extract was mixed with 0.5 mL of 0.1 M borate buffer (pH 8.8) and 0.5 mL of 12 mM L‐phenylalanine. The reaction mixture was incubated, and the change in absorbance was recorded at 290 nm using a UV–visible spectrophotometer at 30‐s intervals for 3 min, starting from zero seconds of incubation. PAL activity was expressed as the change in absorbance per minute per gram of fresh tissue (ΔA_290_ min^−1^ g^−1^ FW) and further quantified as nanomoles of trans‐cinnamic acid formed per minute per g of fresh tissue (nmol trans‐cinnamic acid min^−1^ g^−1^ FW).

### 2.13 | Biocontrol and Plant Growth Promotion Potential of *P. minitans* TNAU‐CM 1 Under Field Conditions

Two field trials were conducted at farmer's fields at Madampatti (trial I) and Sennanur (trial II) villages of Coimbatore district of Tamil Nadu state in India, during May–July and July–September 2022 to evaluate the efficacy of *P. minitans* TNAU‐CM 1 against the pathogen. The experiments were arranged in a Randomised Block Design (RBD) with five treatments and five replications, each plot measuring 20 m^2^. Tropical Giant hybrid cabbage cultivar was planted at a spacing of 30 × 45 cm, with drip irrigation provided throughout the trials. Foliar sprays were applied at 30 DAT during the early head formation stage and again 15 days later at the head fill stage. The treatments included foliar sprays (FS) of *P. minitans* TNAU‐CM 1 stock solution at 5 mL/L (T1), *T. asperellum* TRI 15 stock solution at 5 mL/L (T2), *B. subtilis* Bbv 57 stock solution at 5 mL/L (T3), Tebuconazole + Trifloxystrobin at 1.5 g/L (T4), and a healthy control (T5). All treatments were applied at 30, 45, and 60 DAT. Disease incidence was assessed based on the percentage of infected plants in each plot, and the efficacy of each treatment was expressed as the percentage reduction in disease incidence compared to the untreated control. The percent reduction over control was calculated using,

$$\text{Percent reduction over control}\;\left(\%\right)=C-T/C\times 100$$

where *C* represents the disease incidence in the control and *T* represents the incidence in treated plots. The collected data was subjected to statistical analysis to determine treatment efficacy. Both field trials were conducted within the same geographical region and in a single cropping season to minimise variability in agronomic practices, crop phenology, and baseline disease pressure, thereby ensuring a consistent experimental baseline for treatment comparisons. While this approach strengthens internal validity, environmental metadata such as soil type, rainfall, and pathogen inoculum pressure were not recorded, which may limit the extrapolation of results to other regions or seasons.

### 2.14 Statistical Analysis

SAS software Version 9.2 (SAS Institute, Cary, NC, USA) and XLSTAT software version 19 (XLSTAT, New York, USA) were used to analyse the data. Values are presented as the mean of four replications. For proportion data, a square root or arcsine transformation was applied prior to analysis to meet normality assumptions. Normality and homogeneity of variance were confirmed using the Shapiro–Wilk and Levene's tests, respectively. Data was analysed using one‐way ANOVA in SAS software (Version 9.2; SAS Institute, Cary, NC, USA) and XLSTAT software (Version 19; Addinsoft, New York, USA). Treatment means were compared using Duncan's Multiple Range Test (DMRT) at *p* ≤ 0.05 (Sullivan and Greenland 2013).

## 3 Results

### 3.1 Morphological and Molecular Characterisation of Mycoparasitic Fungal Isolates

Cabbage plants inoculated with the fungal isolate TNAU‐SS‐5 developed necrosis and lesions within 5 days, while control plants remained symptom‐free, confirming the isolate's pathogenicity. Among the 100 collected sclerotia, three were infected by mycoparasites and originated from plants with low levels of pathogen infection. The remaining 97 sclerotia were collected from plants showing moderate (*n* = 35–45) and high (*n* = 41–52) infection levels, none of which exhibited signs of mycoparasitism. Using sclerotia recovered directly from infected plants, rather than inducing them artificially under laboratory conditions, allowed us to assess the natural occurrence of mycoparasitic interactions in the field. A total of 21 mycoparasitic fungal isolates were obtained from the sclerotia of infected cabbage plants. Molecular characterisation using ITS sequence revealed the fungal isolates as *P. minitans* (TNAU‐CM 1, TNAU‐CM 2 and CM 6), *Clonostachys rosea* (TNAU‐CR 01 to TNAU‐CR 05), *Penicillium sclerotiorum* (MF‐8), *Epicoccum nigrum* (MF‐9), *Phoma herbarum* (MF‐10), *Cladosporium cladosporioides* (MF‐11 and MF‐12), *Phoma* sp. (MF‐13 and MF‐14), *Didymella rhei* (MF‐15), *D. glomerata* (MF‐16), *Roussoella neopustulans* (MF‐17), *Talaromyces verruculosus* (MF‐18), and *Chaetomium convolutum* (MF‐19 and MF‐20). The ITS sequences of all identified mycoparasitic fungi were submitted to NCBI with accession numbers, and detailed descriptions of their colony morphology, spore structures, and sequence data are provided in the Supporting Information (Table S1; Figures S1 and S2).

### 3.2 Antifungal Activity of Mycoparasitic Fungus Against *S. sclerotiorum* Under In Vitro Conditions

**FIGURE 1:**
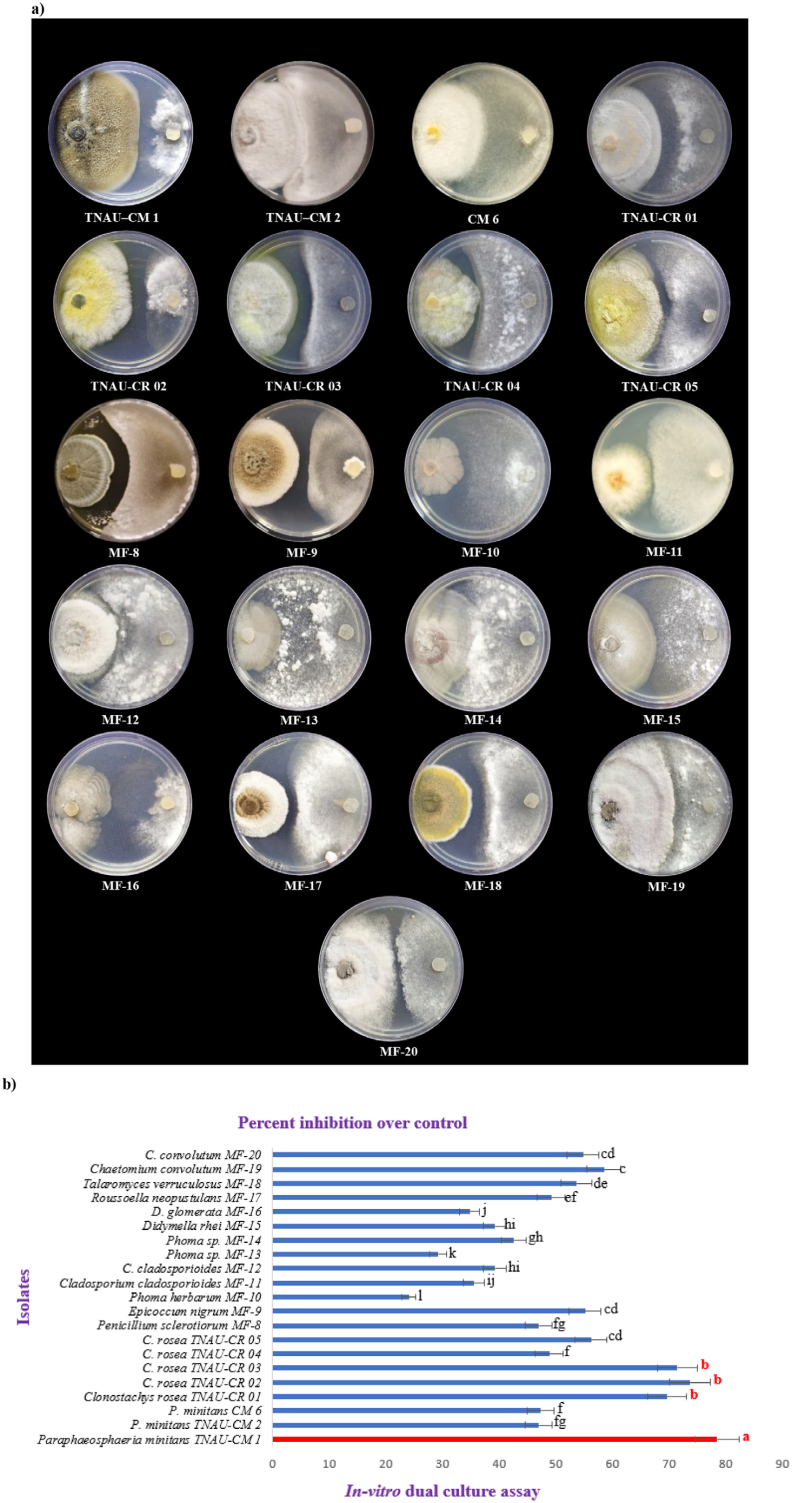
Antagonistic activity of mycoparasitic fungal isolates against *S. sclerotiorum* under in vitro conditions. (a) Dual‐culture assay showing the interaction between mycoparasitic fungi and *S. sclerotiorum*. (b) Antifungal activity expressed as percent inhibition over control for 20 fungal isolates, presented as a bar chart. Values represent the mean ± SE of three replicates per isolate (*n* = 3). The percent inhibition varied significantly among the isolates as analysed by Duncan's Multiple Range Test (DMRT) at *p* ≤ 0.05. TNAU‐CM 1 showed the highest inhibition (78.51%) and was placed in group ‘a’. TNAU‐CR 01 (69.63%), TNAU‐CR 02 (73.70%) and TNAU‐CR 03 (71.48%) were grouped under ‘b’. MF‐19 (58.51%) belonged to group ‘c’, while MF‐20 (54.81%), MF‐9 (55.18%) and TNAU‐CR 05 (56.29%) were grouped under ‘cd’. MF‐18 (53.70%) was placed in ‘de’ and MF‐17 (49.25%) in ‘ef’. TNAU‐CR 04 (48.88%), CM‐6 (47.40%), MF‐8 (47.03%) and TNAU‐CM 2 (47.03%) showed comparable inhibition and were grouped under ‘f’ and ‘fg’. MF‐14 (42.59%) was grouped under ‘gh’, MF‐12 and MF‐15 (39.25%) under ‘hi’, MF‐11 (35.55%) under ‘ij’, and MF‐16 (34.81%) under ‘j’. MF‐13 (29.25%) was placed in group ‘k’, while MF‐10 (24.07%) recorded the lowest inhibition and was grouped under ‘l’. Bars sharing the same letter(s) are not significantly different.

The efficacy of 21 mycoparasitic fungal isolates against *S. sclerotiorum* was evaluated using dual‐culture technique. Among the tested isolates, TNAU‐CM 1 exhibited the highest mycelial growth inhibition at 78.51%, followed by TNAU‐CR 02 (73.70%) and TNAU‐CR 03 (71.48%). In contrast, the lowest inhibition rates were observed in MF‐10 (24.07%) and MF‐13 (29.25%) (Figure 1). Inhibition was assessed after 9 days, when the control plates of *S. sclerotiorum* reached the edge of the Petri dish, thereby providing a fixed endpoint for comparison across isolates. The inhibition percentage was calculated relative to the pathogen's control growth, ensuring normalisation despite differences in growth rates and colony diameters of the tested fungi. These findings indicate that the TNAU‐CM 1 isolate significantly suppresses the mycelial growth *of S. sclerotiorum*, demonstrating strong antagonistic potential. Consequently, TNAU‐CM 1 was selected for further evaluation under glasshouse and field conditions. The identity of TNAU‐CM 1 isolate as *P. minitans*, was further confirmed with the amplification of a 170 bp fragment by the *P. minitans* specific primers Cm spp. 1F and Cm spp. 1R.

### 3.3 | Structural Components of *P. Minitans* TNAU‐CM 1 Against *S. sclerotiorum* Using SEM

**FIGURE 2:**
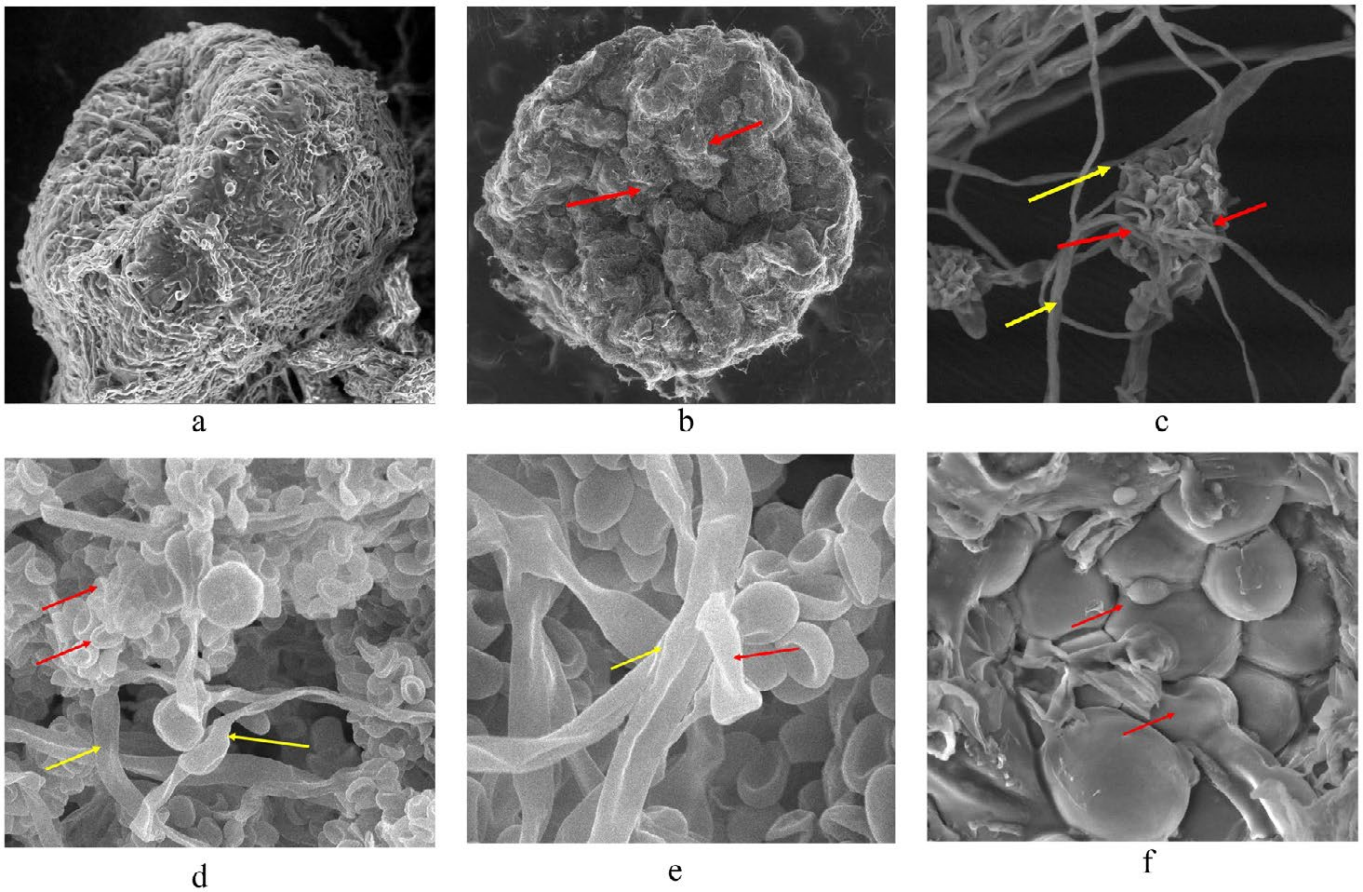
Scanning electron micrographs showing structural components of *S. sclerotiorum* (TNAU‐SS‐5) sclerotia in the absence and presence of *P. minitans* (TNAU‐CM 1). (a) Healthy sclerotium with intact rind (1500×; scale bar, 50 μm). (b) Distorted sclerotium colonised by *P. minitans*, with pycnidiospores visible on the surface (120×; scale bar, 500 μm; red arrows, pycnidiospores). (c) Pycnidiospores of *P. minitans* covering *S. sclerotiorum* hyphae (2000×; scale bar, 50 μm; red arrows, pycnidiospores; yellow arrows, *S. sclerotiorum* hyphae). (d) Aggregate of pycnidiospores on *S. sclerotiorum* hyphae (2000×; scale bar, 10 μm; red arrows, pycnidiospores; yellow arrows, *S. sclerotiorum* hyphae). (e) *P. minitans* hyphae coiling around and distorting *S. sclerotiorum* hyphae (5000×; scale bar, 5 μm; yellow arrows, *S. sclerotiorum* hyphae; red arrows, *P. minitans* hyphae). (f) Appressorium‐like structures formed by *P. minitans* at the sclerotial surface (5000×; scale bar, 10 μm; arrows indicate appressorium‐like structures).

The SEM analysis revealed that TNAU‐CM 1 colonises the sclerotia through an extensive mycelial network and produces pycnidia measuring approximately 2.22 × 1.83 mm (length × width). The pycnidiospores exhibited a smooth surface and were oval to oblong in shape, with dimensions ranging from 6.69 to 7.14 μm in length and 7.82 to 13.46 μm in width. Sclerotia infected by TNAU‐CM 1 displayed significant morphological alterations compared to healthy sclerotial bodies, including a shrivelled, distorted, and shrunken appearance. The hyphal structures of TNAU‐CM 1 demonstrated hypercoiling around and distortion of the pathogenic mycelium, indicating its strong mycoparasitic activity (Figure 2). Additionally, the formation of appressoria‐like structures was observed at the contact points between TNAU‐CM 1 and the sclerotial cortex, suggesting a specialised mechanism for host penetration and colonisation. These structural adaptations further support the mycoparasitic potential of TNAU‐CM 1 in suppressing sclerotial pathogens.

### 3.4 Production of Defence Related Enzymes Produced by *P. minitans* TNAU‐CM 1

The mycoparasitic fungus TNAU‐CM 1 was cultivated in PDB supplemented with different carbon sources to evaluate its enzymatic activity. Among the treatments, xylan‐amended cultures showed the highest endochitinase activity (0.85 ± 0.03 OD at 595 nm), which was significantly higher (*p* < 0.05) than laminarin (0.73 ± 0.02 OD) and CMC (0.68 ± 0.04 OD). Cultures grown in sucrose and chitin‐amended broths exhibited lower endochitinase activity (0.35 ± 0.02 and 0.52 ± 0.03 OD, respectively). The enzyme activity increased from the 7th day after inoculation (DAI), peaked on the 15th DAI, and declined by the 20th DAI. Similarly, endoglucanase activity was highest in chitin‐supplemented cultures (0.79 ± 0.03 OD at 595 nm on 15th DAI), significantly surpassing other treatments (*p* < 0.05). Xylan and laminarin also induced high endocellulase activity, while sucrose and CMC treatments showed minimal enzyme production (0.03 ± 0.01 OD). Endoxylanase activity was notably higher in xylan‐amended broth, peaking at 0.94 ± 0.04 OD on 15th DAI. These results indicate that TNAU‐CM 1 adapts its enzymatic response depending on available carbon sources, with xylan strongly inducing chitinase, chitin promoting glucanase, and laminarin supporting cellulase production (Figure S3). Although cellulase is not directly involved in antagonism against fungus as they lack cellulose, its production may reflect a broader saprophytic or metabolic response of *P. minitans* to complex polysaccharides and antagonism against oomycetes. Notably, laminarin supplementation elicited a more generalised enzymatic response, characterised by elevated cellulase activity while exerting a comparatively limited effect on β‐1,3‐ and β‐1,6‐glucanase production.

### 3.5 Volatile Bioactive Compounds at the Zone of Inhibition of *P. minitans* TNAU‐CM 1 Against *S. sclerotiorum* TNAU‐SS‐5

At the zone of inhibition, GC–MS analysis identified twenty‐four volatile metabolites, including 1,2‐benzenedicarboxylic acid, bis(2‐methylpropyl) ester (7.74%), dibutyl phthalate (6.28%) and 10‐undecynoic acid (4.47%), all known for antimicrobial properties. Other volatile compounds include phthalic acid, hept‐4‐yl isobutyl ester (2.88%), palmitic acid (1.44%), and 9,12‐octadecadienoic acid (Z,Z)‐ and linoleic acid (1.76%) in low concentrations. Although the possibility of minor contributions from the medium or pathogen exists, these comparative controls support the identified VOCs from TNAU‐CM 1. The crude extract of the effective TNAU‐CM 1 isolate demonstrated 54.4% inhibition at 100 ppm concentration, followed by 48.8% and 22.22% inhibition at 75 and 50 ppm. The lowest inhibition was observed at 25 ppm, with an inhibitory percentage of 8.84%, compared to the control.

### 3.6 Molecular Docking Analysis

Experimentally resolved crystal structure is not available for *S. sclerotiorum* SsYCP1, so the structurally characterised cupin protein from *S. cerevisiae* (1ZNP) was used as a surrogate. Pairwise BLASTp alignment showed 81% query coverage and 54.42% sequence identity, with conservation of the canonical β‐barrel cupin domain, supporting its suitability as a homologous model. Homology modelling of SsYCP1 using 1ZNP as the template yielded a reliable structure, further validated by PROCHECK Ramachandran plot analysis, which revealed 96.6% of SsYCP1 residues and 85.4% of 1ZNP residues in the most favoured regions, with no disallowed residues in SsYCP1. Collectively, these validations confirm that 1ZNP is an appropriate and structurally reliable surrogate for SsYCP1 in docking studies (Figure S4). Among the tested compounds, derived from GC–MS, linoleic acid and butyl octyl phthalate exhibited the highest binding affinities, with values of −7.6 and −6.2 kcal/mol, while the fungicidal check compounds Trifloxystrobin and Tebuconazole demonstrated binding affinities of −6.3 and −5.8 kcal/mol, respectively. Molecular visualisation revealed that linoleic acid formed hydrogen bonds with critical residues PHE29, ALA42, TYR44, GLU83, and GLN79 in the target protein 1ZNP. Similarly, butyl octyl phthalate interacted with TRP55, THR86, HIS76, PRO99, ARG98, VAL84, and PRO142 residues, part of the active site of 1ZNP (Table 1). These findings indicate that linoleic acid and butyl octyl phthalate exhibit strong binding interactions with the target protein, suggesting their potential as effective inhibitors. Two major metabolites detected by GC–MS, linoleic acid and butyl octyl phthalate, were procured as pure standards and evaluated for their inhibitory activity against the target pathogenic protein under in vitro conditions. Both compounds exhibited notable inhibition, with linoleic acid showing the highest reduction in protein activity, followed by butyl octyl phthalate. These findings corroborate the molecular docking results, where both compounds displayed strong binding interactions with the target protein, suggesting their potential as effective inhibitors. The molecular interactions of all 14 tested compounds, along with 2D interaction diagrams and comparative analysis, are presented in Figure S5.

**TABLE 1:** Binding affinities and associated interacting residues of secondary metabolites targeting 1ZNP protein.

Compounds name	PubChem ID	1ZNP binding affinity (Kcal/mol)	No. of hydrogen bond	Interacting residues
Benzene, [3‐(2‐cyclohexylethyl)‐6‐cyclopentylhexyl]	279536	−5.3	—	ALA136, TRP140
Dihydroxanthin	536922	−4.9	2	LYS49, ARG52
Phen‐1,4‐diol, 2,3‐dimethyl‐5‐trifluoromethyl	590850	−5.0	2	GLU137, TRP108, ALA136, TRP140
Linolenic acid	5280934	−7.6	2	PHE29, ALA42, TYR44, GLU83, GLN79
Palmitic acid	985	−4.6	3	PRO99, ARG98, VAL84, ILE93, THR86, HIS76
Butyl octyl phthalate	66540	−6.2	3	TRP55, THR86, HIS76, PRO99, ARG98, VAL84, PRO142
Allyl formate	61278	−3.1	—	PRO99, TYR68
1‐beta‐D‐Ribofuranosyl‐2,4(1H,3H)‐pyrimidinedione	6439873	−4.9	2	HIS76, GLU112, ARG52, LEU94
Phthalic acid, isobutyl 2‐pentyl ester	6423867	−4.8	3	SER110, HIS76, TRP55, ARG52
Dibutyl phthalate	3026	−4.7	3	VAL84, PRO142, ILE93, ARG98, HIS76, THR86
Diisobutyl phthalate	6782	−4.6	—	MSE5, ILE10, LEU119, ALA69, TYR68, LEU14, TYR44
1‐Nonadecene	29075	−3.4	—	LEU14, ILE10, LEU119, ALA69, TYR68, TYR67
1‐Docosene	74138	−3.4	3	VAL84, PRO142, THR86, HIS76, ARG98, ILE93
Phthalic acid, hept‐4‐yl isobutyl ester	91720280	−5.0	3	VAL84, PRO142, ARG98, THR86, HIS76
Trifloxystrobin	11664966	−6.3	2	GLU112, HIS76, ARG98, ALA136, TRP55, TRP108
Tebuconazole	86102	−5.8	1	GLY81, VAL84, ALA136, TRP108, TRP55

### 3.7 Molecular Dynamics (MD) Simulations

**FIGURE 3:**
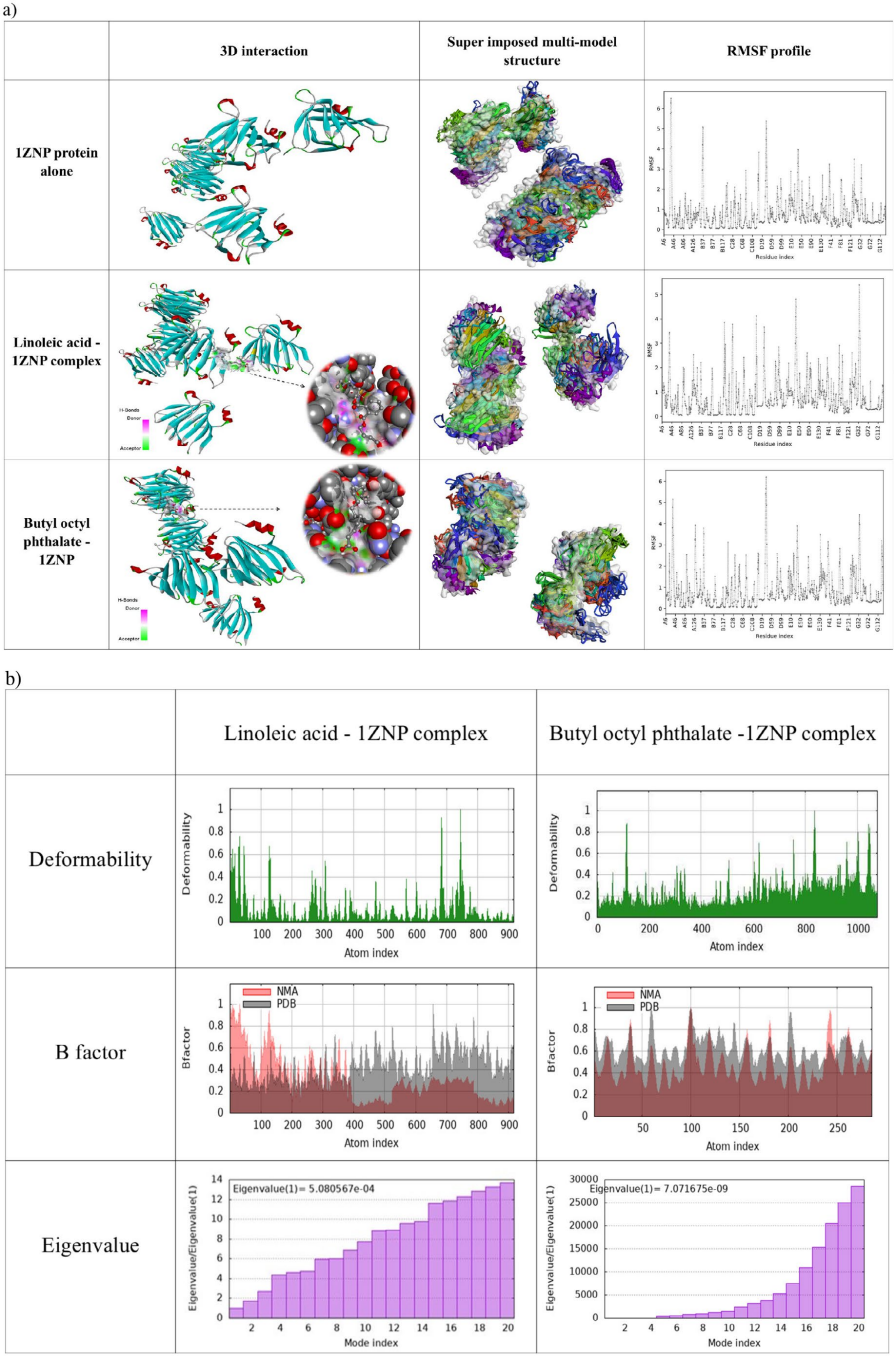
Molecular docking and simulation analysis of 1ZNP with selected ligands. (a) Three‐dimensional interaction maps of 1ZNP alone and in complex with linoleic acid and butyl octyl phthalate, showing interaction views, superimposed multimodal simulated structures, and root mean square fluctuation (RMSF) profiles. (b) Comparative molecular dynamics simulation analysis of linoleic acid–1ZNP and butyl octyl phthalate–1ZNP complexes using iMODS, illustrating deformability, B‐factor plots, and eigenvalues.

**FIGURE 4:**
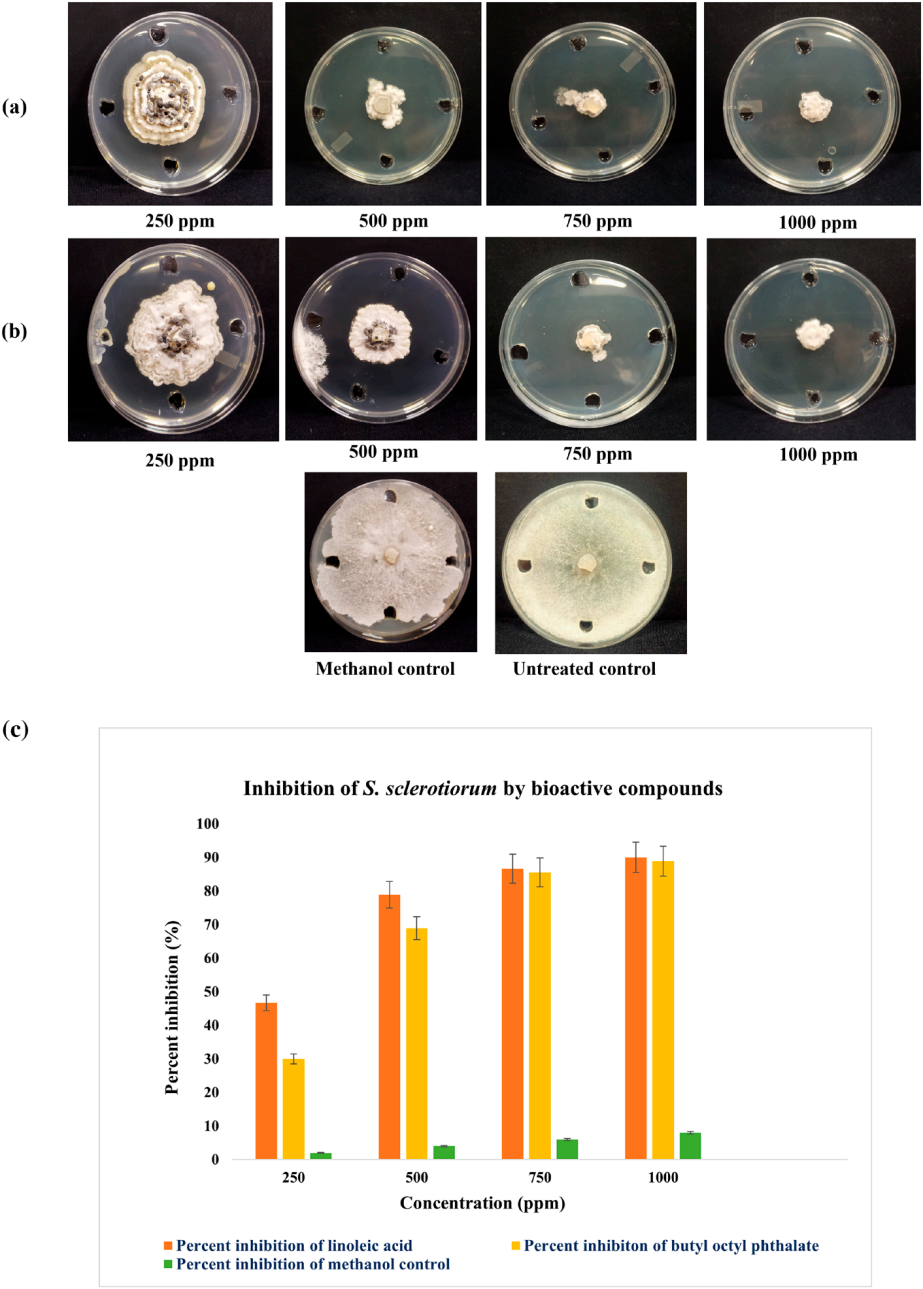
Dose‐dependent antifungal activity of (a) linoleic acid and (b) butyl octyl phthalate against *Sclerotinia sclerotiorum* under in vitro conditions. (c) Bar graph showing the percent inhibition of *S. sclerotiorum* at different concentrations of linoleic acid and butyl octyl phthalate, incorporated to enable comparative visualisation of inhibition percentages in each case. Values represent the mean ± SE of five replications (*n* = 5). Statistical differences among treatments were analysed at *p* ≤ 0.05.

The RMSF values in the unbound 1ZNP protein were observed at residues A35 (6.516 Å), A34 (6.289 Å), A36 (5.815 Å), and A33 (5.742 Å). Upon binding with linoleic acid, these values were significantly reduced to A35 (3.383 Å), A34 (2.606 Å), A36 (3.450 Å), and A33 (1.816 Å), respectively. Similarly, in the butyl octyl phthalate‐1ZNP complex, the RMSF values decreased to A35 (4.775 Å), A34 (3.813 Å), A36 (5.165 Å), and A33 (2.792 Å). These reductions indicate conformational stabilisation, lowering the flexibility of key residues. With the stable average root mean square (RMS) values, the B‐factor analyses confirmed the consistency of flexibility and mobility profiles across both complexes (Figure 3). Eigenvalue analysis revealed 5.08 × 10^−4^ value for the linoleic acid‐1ZNP complex and 7.07 × 10^−4^ for the butyl octyl phthalate‐1ZNP complex, indicating flexible vibrational modes, with increased flexibility observed in regions outside the ligand‐binding sites. Covariance mapping demonstrated coordinated atomic movements in functionally significant regions, with higher activity highlighted in red. The elastic network analysis further reinforced these findings, as dark grey dots on the protein structure corresponded to the highly active regions identified in the covariance maps, suggesting strong ligand‐protein interactions (Figure S6). Wet lab testing using the agar well diffusion method demonstrated that linoleic acid and butyl octyl phthalate inhibited the growth of *S. sclerotiorum*. Linoleic acid showed 46.66%, 78.88%, 86.66% and 90% inhibition at 250, 500, 750 and 1000 ppm, respectively, while butyl octyl phthalate exhibited 30%, 68.88%, 85.55% and 88.88% inhibition at the same concentrations. Wells of 9 mm diameter were used for all treatments. Both compounds significantly reduced mycelial growth compared to the untreated control, with linoleic acid displaying slightly higher antifungal activity than butyl octyl phthalate. The findings validate the *in silico* docking predictions, confirming that these two metabolites exhibit inhibitory activity against the target pathogen, supporting their potential use as biocontrol agents (Figure 4).

### 3.8 Optimization of Suitable Synthetic Broth for Developing Liquid Formulation of *P. minitans* TNAU‐CM 1

Among the four broths, molasses yeast broth demonstrated the highest efficiency in promoting fungal biomass and sporulation, yielding a maximum dry mycelial weight of 16.41 g/L and a spore concentration of 13.21 × 10^8^ spores/mL. This was followed by jaggery yeast broth, which yielded a dry mycelial weight of 13.73 g/L and a spore load of 10.26 × 10^8^ spores/mL. In contrast, malt yeast broth exhibited the lowest fungal growth, with a dry mycelial weight of 11.14 g/L and a sporulation rate of 7.26 × 10^8^ spores/mL (Table S2). The superior performance of molasses yeast broth suggests that it provides an optimal nutrient balance, supporting enhanced fungal biomass production and sporulation. Jaggery yeast broth also proved to be a suitable alternative, though with slightly lower efficiency. Conversely, malt yeast broth was the least effective, possibly due to its limited nutrient composition.

### 3.9 Efficacy of Bioformulation Against Cabbage Head Rot Under Glasshouse Conditions

Foliar application of TNAU‐CM 1 at 5 mL/L on 30, 45, and 60 DAT, which resulted in a disease incidence of 15.1% and is the second most effective measure next to chemical fungicide Tebuconazole + Trifloxystrobin (NATIVO) at 1.5 g/L at 30, 45, and 60 DAT, lowering disease incidence to 5.9% at 60 DAT. In contrast, the untreated control exhibited the highest disease incidence of 22.8% at the same time point. This suggests that TNAU‐CM 1 has potential as an effective biological alternative or supplement to minimise the use of chemical fungicides in managing *S. sclerotiorum* in cabbage plants (Figure S7).

### 3.10 Induced Systemic Resistance

The application of the liquid bioformulation of TNAU‐CM 1 in cabbage plants challenged with *S. sclerotiorum* TNAU‐SS‐5 led to a significant increase in peroxidase (PO), polyphenol oxidase (PPO), and phenylalanine ammonia lyase (PAL) activity at all time intervals, whereas control plants showed minimal variation. Enzyme activity peaked between 3 and 7 DAI before declining across all treatments. The highest peroxidase activity (0.989 min^−1^ g^−1^ at 470 nm) was observed in plants treated with *P. minitans* TNAU‐CM 1 (5 mL/L), followed by *B. subtilis* Bbv 57 (0.952 min^−1^ g^−1^) at 7 DAI. Similarly, PPO activity peaked at 1.513 min^−1^ g^−1^ (495 nm) in *P. minitans*‐treated plants, with *B. subtilis* recording 1.488 min^−1^ g^−1^ at 7 DAI. PAL activity followed the same trend, reaching a maximum of 0.812 min^−1^ g^−1^ (290 nm) in *P. minitans*‐treated plants, followed by *B. subtilis* (0.782 min^−1^ g^−1^) at 7 DAI. Additionally, fungicide‐treated plants showed significant induction of PPO and PAL activity compared to inoculated and uninoculated controls (Figure S8). These findings demonstrate that treatment with TNAU‐CM 1 enhances defence‐related enzyme activities in cabbage, suggesting activation of plant defence responses potentially associated with induced systemic resistance (ISR). However, further studies involving ISR‐related gene expression and Salicylic acid (SA), jasmonate (JA), and ethylene (ET) pathway‐related hormone measurements are needed to confirm ISR induction.

### 3.11 Efficacy of Bioformulation Against Cabbage Head Rot Under Field Conditions

**FIGURE 5:**
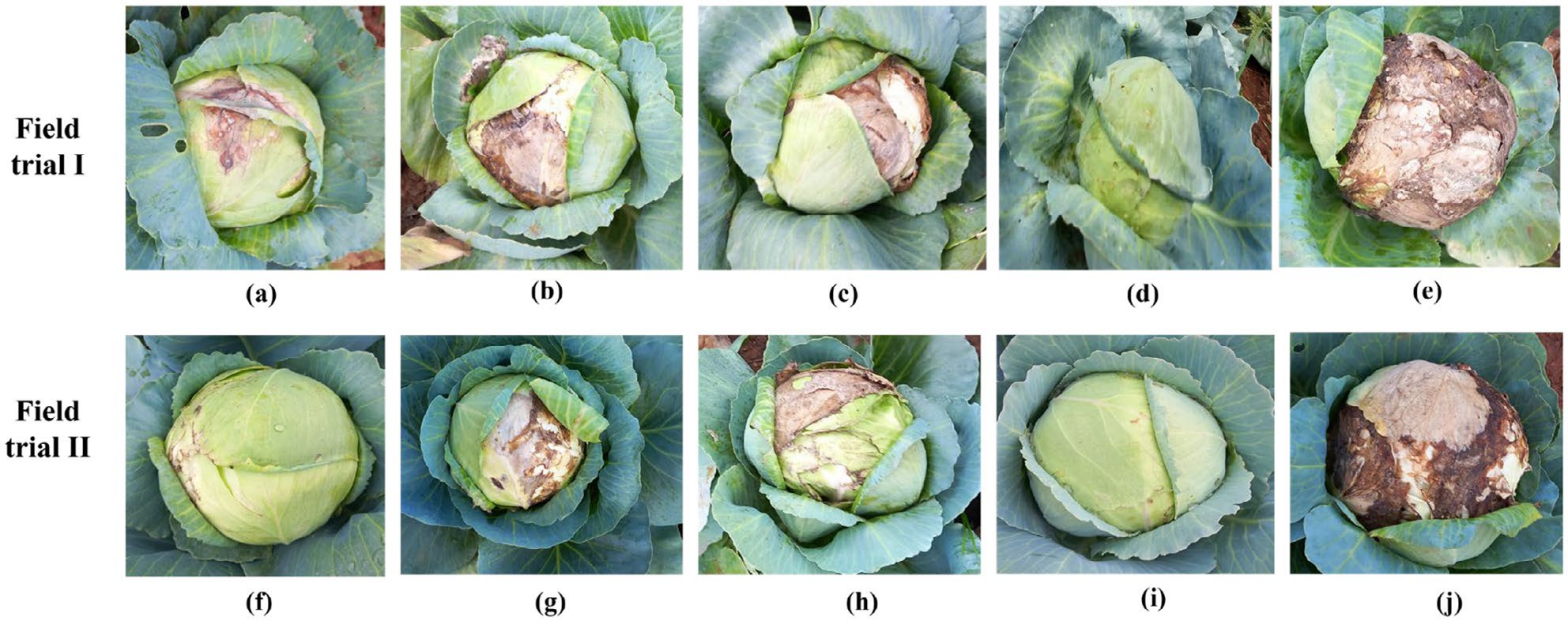
Efficacy of biocontrol agents on the severity of cabbage head rot pathogen under field conditions. Representative cabbage heads were collected at the end of the cropping season (≈90 days after transplanting) following foliar applications at 30, 45, and 60 DAT in two field trials conducted in Coimbatore district, Tamil Nadu. Field trial I—Madampatti: (a) *P. minitans* TNAU‐CM 1 at 5 mL/L, (b) *T. asperellum* (TRI 15) at 5 mL/L, (c) *B. subtilis* (Bbv 57) at 5 mL/L, (d) chemical fungicide Tebuconazole + Trifloxystrobin (NATIVO) at 1.5 g/L, and (e) untreated control. Field trial II—Sennanur: (f) *P. minitans* TNAU‐CM 1 at 5 mL/L, (g) *T. asperellum* (TRI 15) at 5 mL/L, (h) *B. subtilis* (Bbv 57) at 5 mL/L, (i) Tebuconazole + Trifloxystrobin (NATIVO) at 1.5 g/L, and (j) untreated control. Representative plants shown correspond to the median disease incidence for each treatment.

**FIGURE 6:**
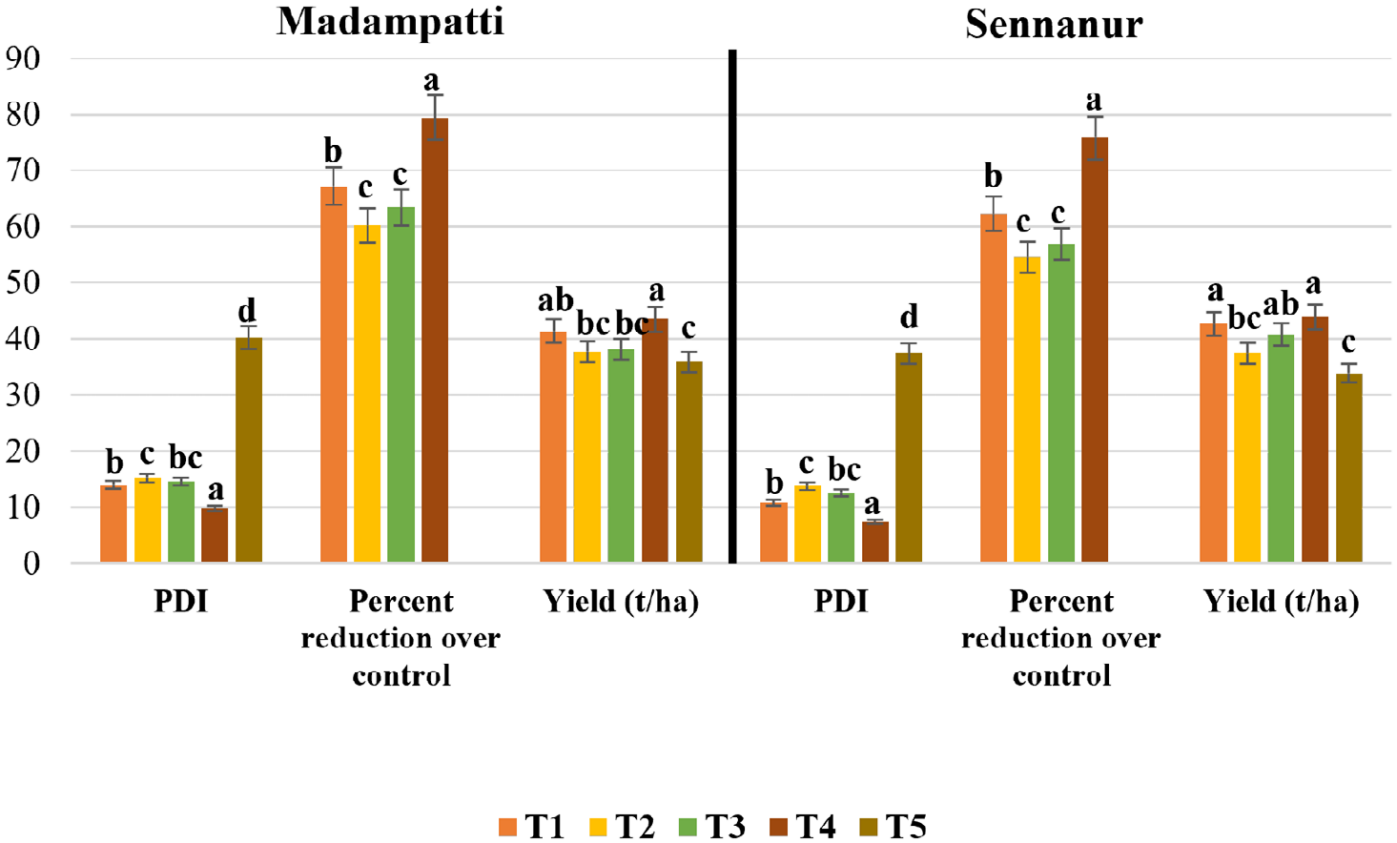
Efficacy of biocontrol agents on the severity of cabbage head rot pathogen under field trial I (Madampatti) and II (Sennanur). Data represent mean ± SE of three replications. Percent disease incidence was subjected to two‐way ANOVA (treatment × time), and means were separated using Tukey's HSD test (*p* ≤ 0.05). Different letters above bars indicate significant differences among treatments at each time point. Treatment T1—Foliar spray of *P. minitans* (TNAU‐CM 1) at 5 mL/L, T2—Foliar spray of *T. asperellum* (TRI 15) at 5 mL/L, T3—Foliar spray of *B. subtilis* (Bbv 57) at 5 mL/L, T4—Foliar spray of Tebuconazole + Trifloxystrobin at 1.5 g/L and T5—Inoculated control without any treatments.

In both the field trials, application of fungicide Tebuconazole + Trifloxystrobin (NATIVO) at 1.5 g/L, at 30, 45, and 60 DAT, proved to be the most effective, recording low disease incidence of 9.77% and 7.39% (trial I and II) and high percentage (67.72 and 75.86) of reduction over control. This percentage reduction over control represents the biocontrol efficiency (BCE), calculated as: BCE (%) = [(Disease incidence in control − Disease incidence in treatment)/Disease incidence in control] × 100. Among the biocontrol agents, *P. minitans* TNAU‐CM 1 was the most effective in both trials, with a disease incidence of 13.99% and 10.84% and with percentage reduction as 67.72% and 62.27% over control, followed by *B. subtilis* Bbv 57, which recorded a 14.57% and 12.57% incidence and 63.39% and 56.88% reduction. Yield was highest in the fungicide treatment (43.51 and 43.95 tons/ha), followed by *P. minitans* TNAU‐CM 1 (41.37 and 42.68 tons/ha). Both *B. subtilis* Bbv 57 (38.15 and 40.76 tons/ha) and *T. asperellum* TRI 15 (37.76 and 37.44 tons/ha) yielded similarly and were on par with *P. minitans* TNAU‐CM 1. The control treatment recorded the lowest yield at 35.9 and 33.88 tons/ha (Figures 5 and 6).

## 4 Discussion

*S. sclerotiorum*, the causal agent of head rot, is a highly challenging plant pathogen due to its extensive host array, high virulence, and long persistence in soil through sclerotia. It infects over 400 plant species, making crop rotation unproductive, while its destructive infection mechanisms, including production of oxalic acid and cell wall‐degrading enzymes, enable rapid colonisation of multiple plant species (Bolton et al. 2006). Sclerotia can endure in soil for more than 10 years, battling chemical, physical, and biological degradation. Chemical fungicides like carbendazim, boscalid, NATIVO, and fluazinam have shown efficacy; however, their frequent use will lead to resistance in *S. sclerotiorum*, necessitating the development of sustainable management strategies (Tao et al. 2021). Since sclerotia are the primary survival structures of the pathogen, current research has focused on exploiting and characterising mycoparasites that primarily target and degrade the sclerotial structure.

In the search for mycoparasites, only three out of the 100 collected sclerotia displayed visible signs of mycoparasitism. This suggests that natural mycoparasitic infections are relatively rare under field conditions, possibly due to environmental factors, competition with other soil microbes, or variations in the susceptibility of sclerotia to fungal colonisation. Abiotic factors like temperature, moisture, and soil composition (Hamid et al. 2013) and competition among soil microbes (Partridge et al. 2006; Li et al. 2010) play a vital role in establishing mycoparasites in field environments. Among the 21 mycoparasitic fungal isolates, TNAU‐CM 1, belonging to *P. minitans*, exhibited the highest pathogen inhibition (78.51%), demonstrating it as the ideal candidate for managing head rot in cabbage. Earlier studies have also found *P. minitans* as the vital agent in managing *Sclerotinia* stem rot disease in *Brassica* species (Yang et al. 2007; Wang et al. 2024). Collectively, these findings affirm the strong antagonistic potential of *P. minitans* TNAU‐CM1 and provide a foundation for exploring its enzyme production and metabolite‐mediated antifungal mechanisms.

In the enzyme activity profiling, *P. minitans* TNAU‐CM 1 isolate showed higher endochitinase and endocellulase activity, highlighting cell wall degradation as part of its hyperparasitic mechanism (Yang et al. 2022). Hydrolytic enzymes are pivotal for *P. minitans* parasitism, facilitating degradation of the sclerotial cell wall (Lou et al. 2015; Zhao et al. 2020). Disruption of the chitinase genes *CmCH1* and *CmCH10* markedly reduces sclerotial colonisation, confirming their essential role in mycoparasitic efficacy (Wang et al. 2025). Similarly, elevated β‐1,3‐glucanase and xylanase activities have been linked to the degradation of sclerotial cell walls and enhanced parasitic efficiency in *P. minitans* (Giczey et al. 2001). Subsequently, in GC–MS analysis, 24 bioactive metabolites, including n‐hexadecanoic acid, phthalic acid derivatives, and 9,12‐octadecadienoic acid, known previously for antifungal activity were identified (Jayaprakashvel and Mathivanan 2011; Derbalah et al. 2012). Similarly, Tomprefa et al. (2009) demonstrated that macrosphelide A, a metabolite produced by *P. minitans*, significantly suppressed the growth of *S. sclerotiorum*, reinforcing its role in fungal inhibition. The agar well diffusion assay in our study confirmed the inhibitory effect of metabolites obtained from *P. minitans* cultured in pure PDB medium, ensuring that the crude extracts did not contain compounds produced by *S. sclerotiorum*. This approach allowed us to attribute the antifungal activity solely to the mycoparasite's metabolites, independent of any potential contributions from the pathogen. Comparably, Sun et al. (2015) and Yang et al. (2020) reported that *P. minitans* metabolites disrupted *S. sclerotiorum* hyphal integrity, highlighting their role in mycoparasitism. By integrating enzyme assays with metabolite profiling, we demonstrate that both enzymatic degradation and chemical interference act synergistically to restrict the pathogen's development.

In the molecular docking analysis, metabolites linoleic acid and butyl octyl phthalate exhibited the highest binding affinities to 1ZNP, a reference protein structurally similar to the SsYCP1, a putative effector molecule of *S. sclerotiorum*. This suggests that these compounds may strongly interact and potentially inhibit the virulence protein, thereby reducing the pathogen's aggressiveness. Earlier, linoleic acid has been reported to inhibit fungal growth by disrupting membrane integrity, while phthalate derivatives have shown strong antimicrobial activity against phytopathogenic fungi (Fan et al. 2021). Further, the root mean square fluctuation (RMSF) analysis demonstrated a significant reduction in flexibility at key residues (A33–A36) of 1ZNP protein upon binding with linoleic acid and butyl octyl phthalate. These findings are consistent with previous studies highlighting the stabilising effect of bioactive metabolites on fungal virulence proteins. Correspondingly, Fatma et al. (2021) observed that fatty acid derivatives reduced RMSF values in fungal effectors, leading to structural rigidity and reduced enzymatic activity. Similarly, Chen et al. (2023) reported that phthalate esters significantly restricted the conformational flexibility of fungal proteins, thereby impairing their function. Importantly, while SsYCP1 represents a specific virulence factor *in S. sclerotiorum*, linoleic acid and phthalate derivatives are known to act more broadly by disrupting membrane integrity and protein conformation in other fungal pathogens as well. This suggests that the inhibitory effect may not be entirely species‐specific but could extend to fungi with structurally conserved effector proteins, thereby enhancing their potential as broad‐spectrum antifungal agents. However, further comparative studies across diverse fungi are needed to confirm the degree of selectivity. Thus, our computational results not only validate the bioactivity of *P. minitans* metabolites but also provide a mechanistic explanation that connects metabolite activity to pathogen virulence suppression, bridging in vitro and *in silico* findings.

For mass multiplication of potential biocontrol agents, molasses‐yeast broth supported optimal *P. minitans* sporulation, yielding the highest dry mycelial weight (16.41 g/L) and spore production (13.21 × 10^8^ spores/mL). This aligns with Chitrampalam et al. (2011), who reported superior biomass and conidial production in molasses‐yeast broth than in PDB. Similar findings have been observed in other biocontrol fungi, as reported by Abdenaceur et al. (2022), where *Trichoderma* species exhibited enhanced sporulation in nutrient‐rich media, while Lee et al. (2012) demonstrated that carbohydrate‐based substrates improved fungal biomass accumulation. In this study, *B. subtilis* Bbv 57 and *T. asperellum* TRI 15 were selected as reference microbial agents because they are among the most commercially available and well‐established biocontrol strains and widely adopted in the market. This allowed benchmarking *P. minitans* against microbial standards with proven field efficacy and farmer acceptance, thereby ensuring the comparison between biocontrol agents. The inhibitory activity of these microorganisms is attributable to the diverse antifungal compounds they produce (Deshmukh et al. 2018). *Trichoderma* spp. is known to secrete cell wall–degrading enzymes (chitinases, glucanases, proteases) and secondary metabolites such as gliotoxin and peptaibols, while *B. subtilis* strains produce lipopeptides (iturin, fengycin, and surfactin) with both antifungal and plant growth–promoting properties (Hamrouni et al. 2021; Saiyam et al. 2024). By contrast, *P. minitans* primarily acts as a mycoparasite on sclerotia, producing hydrolytic enzymes and metabolites that suppress *S. sclerotiorum* survival and multiplication. Although *T. asperellum* TRI 15 and *B. subtilis* Bbv 57 showed field efficacy comparable to *P. minitans* TNAU‐CM 1, their mechanisms differ. *P. minitans* is highly specialised in parasitising sclerotia, while *Trichoderma* acts through mycoparasitism and antifungal metabolites, and *Bacillus* through lipopeptides and induced resistance. Thus, all three agents reduce pathogen viability but via distinct strategies. Targeting sclerotia provides a consistent intervention point, as these structures ensure pathogen persistence. While *P. minitans* directly colonises and degrades sclerotia, *Trichoderma* and *Bacillus* may only disrupt them indirectly. This highlights sclerotia parasitism as a sustainable approach against several sclerotia‐forming fungi, though integration with complementary microbes is essential for broader protection.

Further, our study demonstrated that foliar application of *P. minitans* TNAU‐CM 1 significantly reduced *S. sclerotiorum* incidence in cabbage. This aligns with Jones et al. (2014), who found that *P. minitans* LU112 reduced disease incidence by 59% under pot conditions. Similarly, Gerlagh et al. (2004) reported a 50% reduction in soil‐applied treatments, while Reich et al. (2017) observed suppression of sclerotinia blight in alfalfa. The effect of *P. minitans* TNAU‐CM 1 against *S. sclerotiorum* is also confirmed through two field trials where over 60% reduction in the head rot disease was observed. Previously, Jones et al. (2014) reported a 59% disease reduction under controlled conditions using *P. minitans* LU112. However, it should be noted that the present study was conducted under limited environmental conditions and locations, which may influence the generalisability of the findings. This limitation has been acknowledged, and future research is planned as multi‐location, multi‐season trials with comprehensive environmental data collection to enhance the external validity and applicability of *P. minitans* TNAU‐CM 1 in diverse agro‐climatic zones.

Overall, this study demonstrates that the efficacy of *P. minitans* TNAU‐CM1 against *S. sclerotiorum* is supported by a consistent chain of evidence, spanning laboratory inhibition assays, enzyme and metabolite analyses, computational protein–ligand interactions, and field‐level disease suppression. By connecting these experimental layers, our work not only validates *P. minitans* as a reliable biocontrol agent but also provides an integrated framework for evaluating microbial antagonists from mechanism to field performance. This holistic perspective positions *P. minitans* TNAU‐CM1 as a field‐ready alternative for sustainable management of cabbage head rot.

## 5 Conclusion

Our study demonstrates *P. minitans* TNAU‐CM 1 as an effective biocontrol agent against *S. sclerotiorum*, the causative pathogen of cabbage head rot. Through morphological, molecular, and SEM analyses, TNAU‐CM 1 demonstrated strong mycoparasitic activity, characterised by direct colonisation and degradation of sclerotia via pycnidia formation, hypercoiling, and appressoria‐like structures for host penetration. GC–MS profiling identified 24 bioactive metabolites, with linoleic acid and butyl octyl phthalate exhibiting strong antifungal properties. Molecular docking and dynamics simulations further confirmed the stable interactions of these compounds with pathogenic proteins, with superior binding affinities compared to commercial fungicides and suggested a potential mode of action by disrupting fungal virulence. The use of molasses yeast broth as an economical medium enhances its scalability for large‐scale applications. This work integrates metabolite profiling, *in silico* interaction analysis, and multi‐season field validation to establish the practical biocontrol potential of *P. minitans* TNAU‐CM 1. Field trials validated the efficacy of *P. minitans* TNAU‐CM 1, achieving a 67.72% reduction in disease, with yield levels comparable to those of chemical fungicides. These findings reinforce the potential of *P. minitans* TNAU‐CM 1 as an important biocontrol agent for managing cabbage head rot and promoting eco‐friendly crop protection strategies.

## Author Contributions

**Meena V. Ruppavalli:** performed the lab experiments, data curation, writing – original draft, writing – review and editing; **Muthusamy Karthikeyan:** conceptualization, methodology, investigation, resources, supervision, project administration; **Iruthayasamy Johnson:** conceptualization, methodology, investigation, resources, supervision, project administration; **Sambasivam Periyannan:** conceptualization, supervision, funding, reviewing and editing; **Parthiban V. Kumaresan:** formal analysis, validation, writing and review and editing; **Balakrishnan Prithiviraj:** formal analysis, validation, writing and review and editing; **Sivaji Jeevanantham:** methodology, writing – review and editing, investigation, supported in bioinformatics data.

## Funding

This work was supported by MITACS‐SICI Partnership, IT23781.

## Conflicts of Interest

The authors declare no conflicts of interest.

## Supporting information


**Figure S1:** Morphological characterisation of 21 mycoparasitic fungal isolates. *P. minitans* TNAU‐CM 1 (OL614782), TNAU‐CM 2 (OL614980) and CM 6 (ON025056); *C. rosea* TNAU‐CR 01 (MZ754407), TNAU‐CR 02 (ON025052), TNAU‐CR 03 (ON025053), TNAU‐CR 04 (ON025054) and TNAU‐CR 05 (ON025055); *P. sclerotiorum* MF‐8 (ON062087); *E. nigrum* MF‐9 (ON024790); *P. herbarum* MF‐10 (ON062193); *C. cladosporioides* MF‐11 (ON045141) and MF‐12 (ON024886); *Phoma* sp. MF‐13 (ON025047) and MF‐14 (ON025046); *D. rhei* MF‐15 (ON025049); *D. glomerata* MF‐16 (ON025050); *R. neopustulans* MF‐17 (ON025051), *T. verruculosus* MF‐18 (ON025057); *C. convolutum* MF‐19 (ON025068) and MF‐20 (ON025067).
**Figure S2:** Phylogenetic characterisation of different mycoparasites. (a) *C. minitans*, (b), *P. minitans*, (c) *C. rosea* , (d) *C. cladosporioides*, (e) *P. sclerotiorum*, (f) *E. nigrum* , (g) *Phoma* sp., (h) *D. glomerata* and *D. rhei*, (i) *R. neopustulans*, (j) *T. verruculosus* , (k) *C. convolutum*.
**Figure S3:** Enzymatic activity of *P. minitans* TNAU‐CM 1 grown in PDB supplemented with different carbon sources, recorded at 7th, 10th, 15th, and 20th days after inoculation (DAI). (a) Endochitinase activity, (b) Endoglucanase activity, (c) Endocellulase activity, and (d) Endoxylanase activity. Different carbon sources were used to evaluate their role in inducing specific enzyme systems. Time intervals were selected to capture the dynamics of enzyme production. Values represent the mean ± SE of three replications; Different letters above bars indicate significant differences at *p* < 0.05.
**Figure S4:** Ramachandran plot validation of protein structures. Ramachandran plots showing stereochemical quality of (a) 1ZNP template protein and (b) modelled SsYCP1. The majority of residues lie in the most favoured regions, with no residues in disallowed regions for SsYCP1, confirming the structural reliability of both models for docking analysis.
**Figure S5:** Molecular docking interactions (2D) of secondary metabolites from TNAU‐CM 1 with the 1ZNP protein. (a) Benzene, [3‐(2‐cyclohexylethyl)‐6‐cyclopentylhexyl]. (b) Dihydroxanthin. (c) Phen‐1,4‐diol, 2,3‐dimethyl‐5‐trifluoromethyl. (d) Linolenic Acid. (e) Palmitic Acid. (f) Butyl octyl phthalate. (g) Allyl formate. (h) 1‐beta‐D‐Ribofuranosyl‐2,4(1H,3H)‐pyrimidinedione. (i) Phthalic acid, isobutyl 2‐pentyl ester. (j) Dibutyl Phthalate. (k) Diisobutyl phthalate. (l) 1‐Nonadecene. (m) 1‐Docosene. (n) Phthalic acid, hept‐4‐yl isobutyl ester. (o) Trifloxystrobin. (p) Tebuconazole.
**Figure S6:** MD simulation of linoleic acid‐1ZNP and butyl octyl phthalate‐1ZNP complexes. (a, b) covariance map and elastic network model of linoleic acid‐1ZNP; (c, d) covariance map and elastic network model of bansutyl octyl phthalate‐1ZNP.
**Figure S7:** Efficacy of bioformulation against cabbage head rot under greenhouse conditions. T1—Foliar spray of *P. minitans* (TNAU‐CM 1) stock at 5 mL/L, T2—Foliar spray of *T. asperellum* (TRI 15) at 5 mL/L, T3—Foliar spray of *B. subtilis* (Bbv 57) at 5 mL/L, T4—Foliar spray of Tebuconazole + Trifloxystrobin at 1.5 g/L and T5—Inoculated control without any treatments. Data represent mean ± SE of three replications. Percent disease incidence was subjected to two‐way ANOVA (treatment×time), and means were separated using Tukey's HSD test (*p* ≤ 0.05). Different letters above bars indicate significant differences among treatments at each time point.
**Figure S8:** Induction of defence‐related enzymes in cabbage treated With *P. minitans* TNAU‐CM 1. T1—Foliar spray of *P. minitans* (TNAU‐CM 1) at 5 mL/L, T2—Foliar spray of *T. asperellum* (TRI 15) at 5 mL/L, T3—Foliar spray of *B. subtilis* (Bbv 57) at 5 mL/L, T4—Foliar spray of Tebuconazole + Trifloxystrobin at 1.5 g/L, T5—Inoculated control without any treatments, and T6—Healthy uninoculated control.

**Table S1:** Morphological and molecular characterisation of mycoparasitic fungal isolates.
**Table S2:** Population of *P. minitans* (TNAU‐CM 1) in different liquid medium on 25th DAI.

## Data Availability

The data that supports the findings of this study are available in the Supporting Information of this article.
