# Supplementary material for: Exploiting Paraphaeosphaeria minitans and Its Antifungal Metabolites as Bio‐Fungicides for Eco‐Friendly Management of Head Rot Disease in Cabbage
Source: Microb Biotechnol. 2026 Jan 30;19(2):e70309. doi: 10.1111/1751-7915.70309 (PMC12859388; doi:10.1111/1751-7915.70309)
Supplement: Supplementary file 1 — Figure S1: Morphological characterisation of 21 mycoparasitic fungal isolates. P. minitans TNAU‐CM 1 (OL614782), TNAU‐CM 2 (OL614980) and CM 6 (ON025056); C. rosea TNAU‐CR 01 (MZ754407), TNAU‐CR 02 (ON025052), TNAU‐CR 03 (ON025053), TNAU‐CR 04 (ON025054) and TNAU‐CR 05 (ON025055); P. sclerotiorum MF‐8 (ON062087); E. nigrum MF‐9 (ON024790); P. herbarum MF‐10 (ON062193); C. cladosporioides MF‐11 (ON045141) and MF‐12 (ON024886); Phoma sp. MF‐13 (ON025047) and MF‐14 (ON025046); D. rhei MF‐15 (ON025049); D. glomerata MF‐16 (ON025050); R. neopustulans MF‐17 (ON025051), T. verruculosus MF‐18 (ON025057); C. convolutum MF‐19 (ON025068) and MF‐20 (ON025067). Figure S2: Phylogenetic characterisation of different mycoparasites. (a) C. minitans, (b), P. minitans, (c) C. rosea , (d) C. cladosporioides, (e) P. sclerotiorum, (f) E. nigrum , (g) Phoma sp., (h) D. glomerata and D. rhei, (i) R. neopustulans, (j) T. verruculosus , (k) C. convolutum. Figure S3: Enzymatic activity of P. minitans TNAU‐CM 1 grown in PDB supplemented with different carbon sources, recorded at 7th, 10th, 15th, and 20th days after inoculation (DAI). (a) Endochitinase activity, (b) Endoglucanase activity, (c) Endocellulase activity, and (d) Endoxylanase activity. Different carbon sources were used to evaluate their role in inducing specific enzyme systems. Time intervals were selected to capture the dynamics of enzyme production. Values represent the mean ± SE of three replications; Different letters above bars indicate significant differences at p < 0.05. Figure S4: Ramachandran plot validation of protein structures. Ramachandran plots showing stereochemical quality of (a) 1ZNP template protein and (b) modelled SsYCP1. The majority of residues lie in the most favoured regions, with no residues in disallowed regions for SsYCP1, confirming the structural reliability of both models for docking analysis. Figure S5: Molecular docking interactions (2D) of secondary metabolites from TNAU‐CM 1 with the 1ZNP protein. [file MBT2-19-e70309-s001.doc]

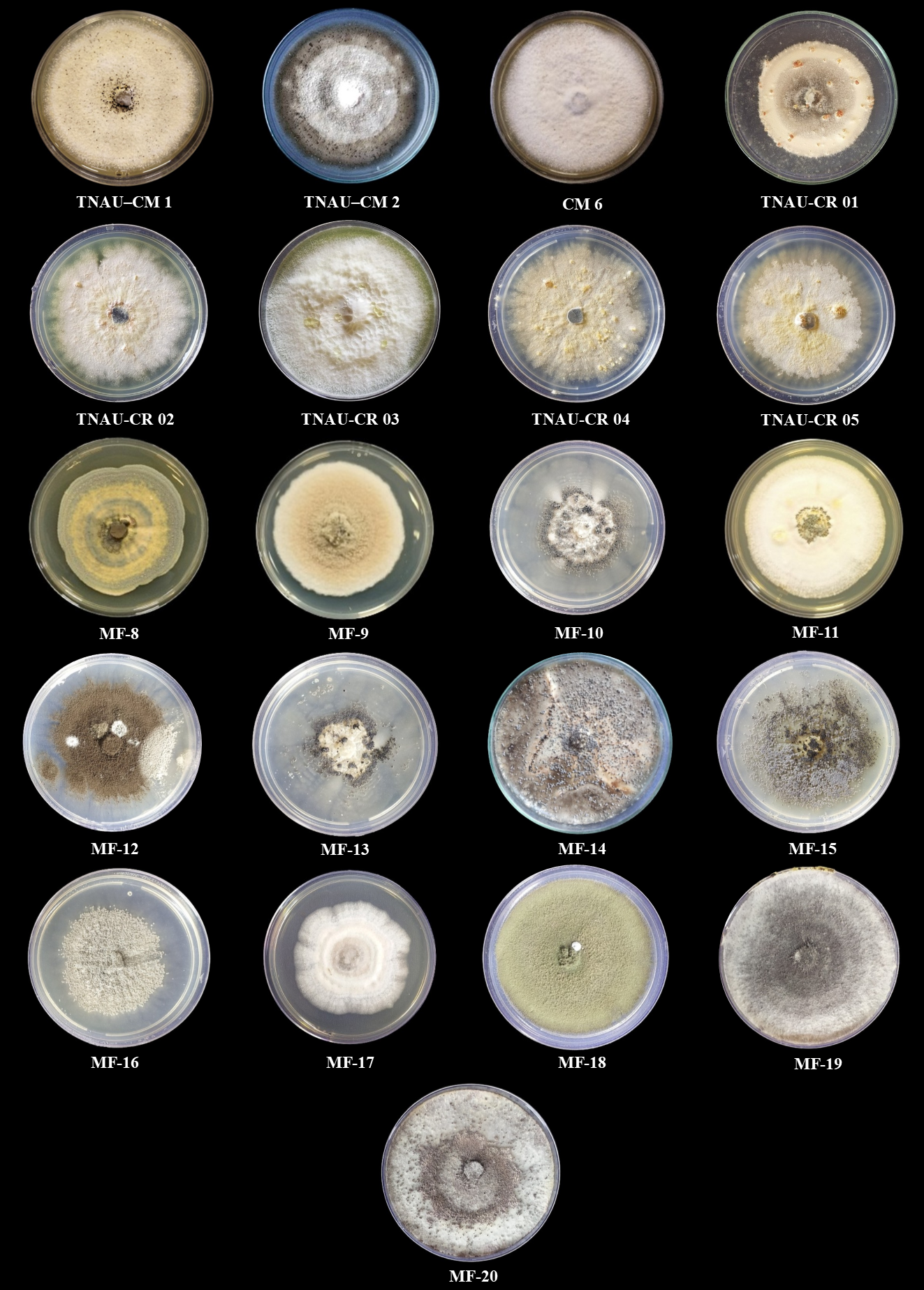


**SUPPLEMENTARY FIGURE S1** | Morphological characterization of 21 mycoparasitic fungal isolates*.* *P.* *minitans* TNAU-CM 1 (OL614782), TNAU-CM 2 (OL614980) and CM 6 (ON025056); *C. rosea* TNAU-CR 01 (MZ754407), TNAU-CR 02 (ON025052), TNAU-CR 03 (ON025053), TNAU-CR 04 (ON025054) and TNAU-CR 05 (ON025055); *P. sclerotiorum* MF-8 (ON062087); *E. nigrum* MF-9 (ON024790); *P. herbarum* MF-10 (ON062193); *C. cladosporioides* MF-11 (ON045141) and MF-12 (ON024886); *Phoma sp.* MF-13 (ON025047) and MF-14 (ON025046); *D. rhei* MF-15 (ON025049); *D. glomerata* MF-16 (ON025050); *R. neopustulans* MF-17 (ON025051), *T. verruculosus* MF-18 (ON025057); *C. convolutum* MF-19 (ON025068) and MF-20 (ON025067).

| 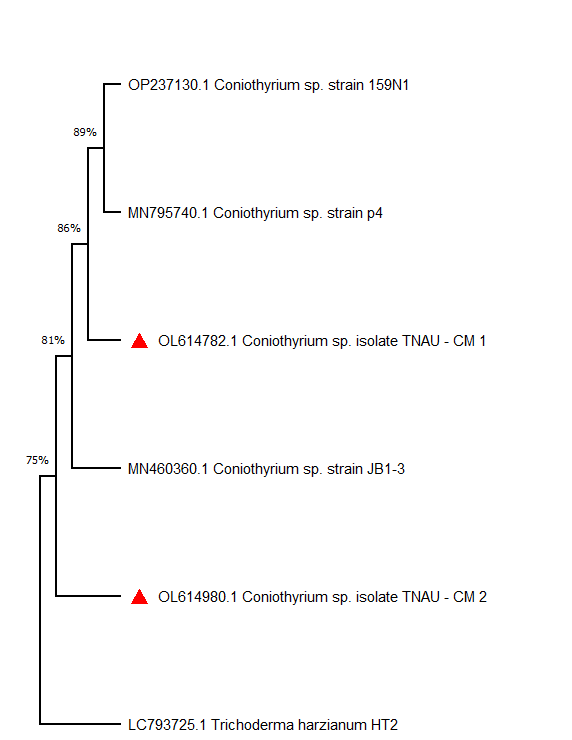**a)** | 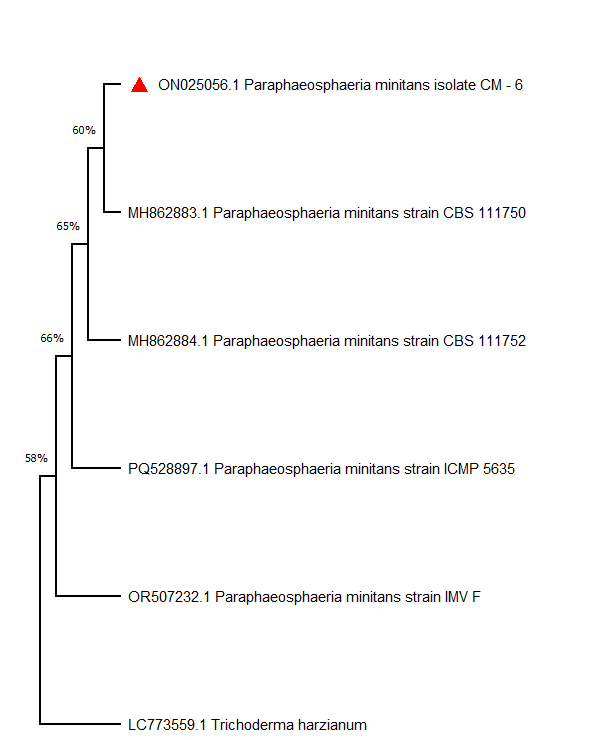**b)** |
| --- | --- |
| 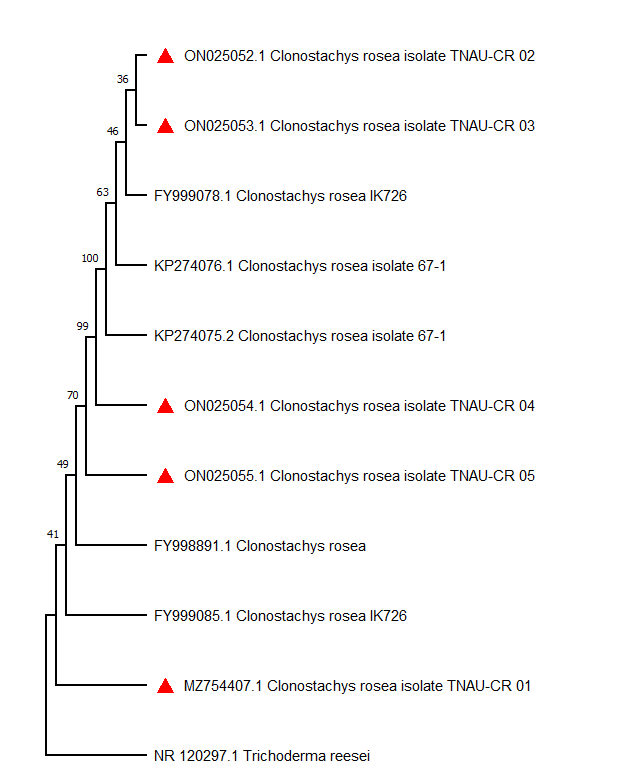**c)** | 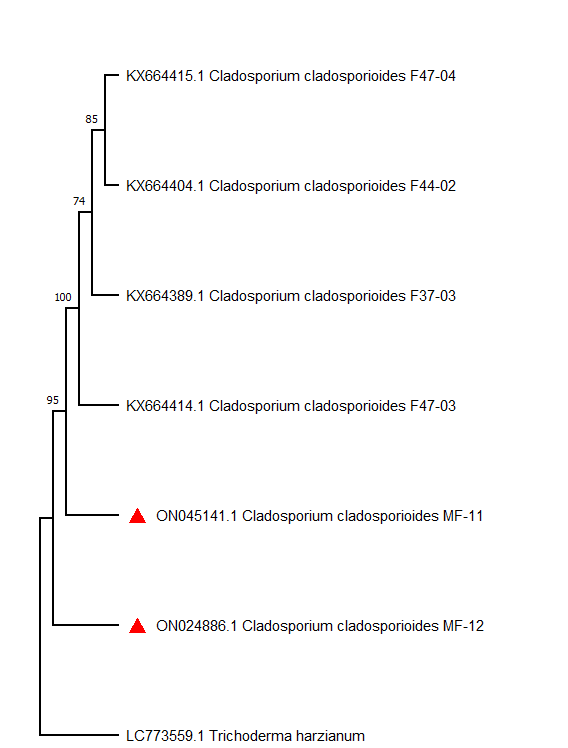**d)** |

| 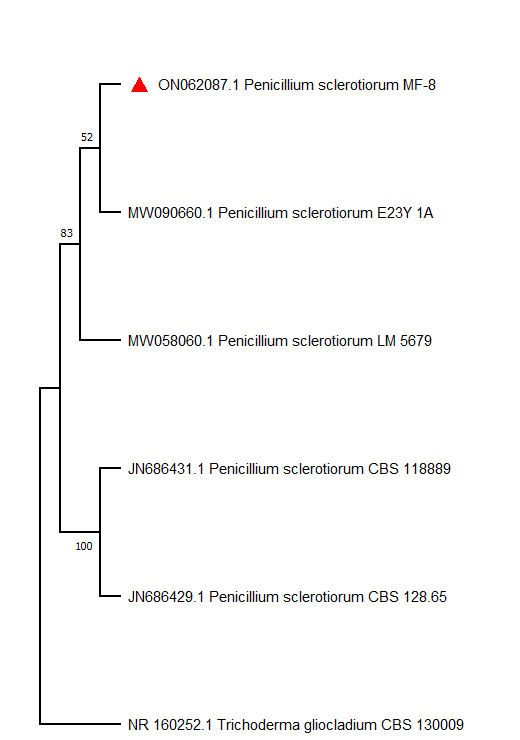**e)** | 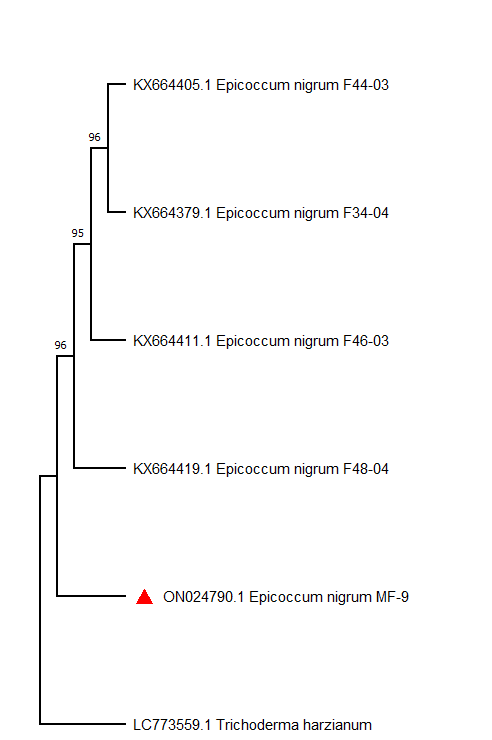**f)** |
| --- | --- |
| 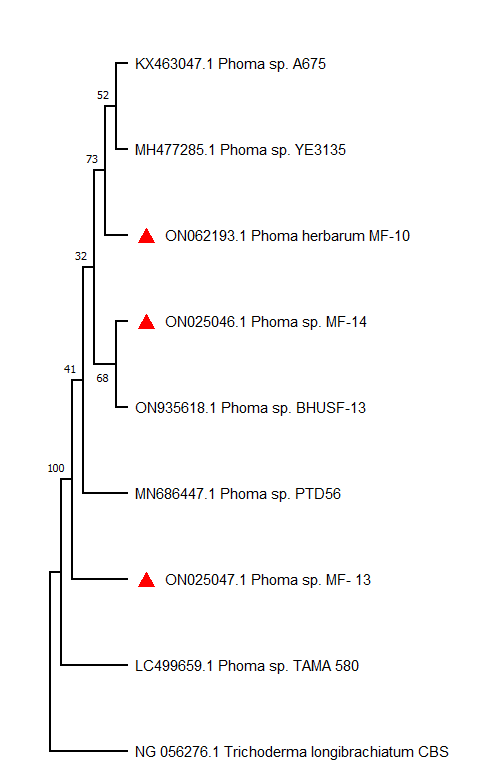**g)** | 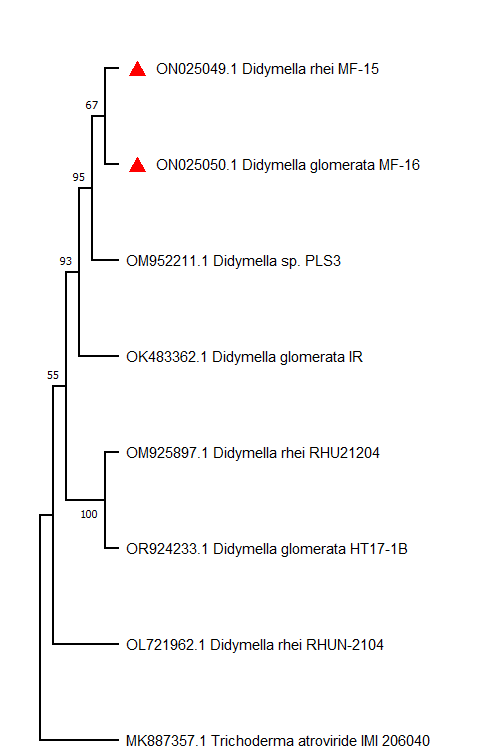**h)** |

| 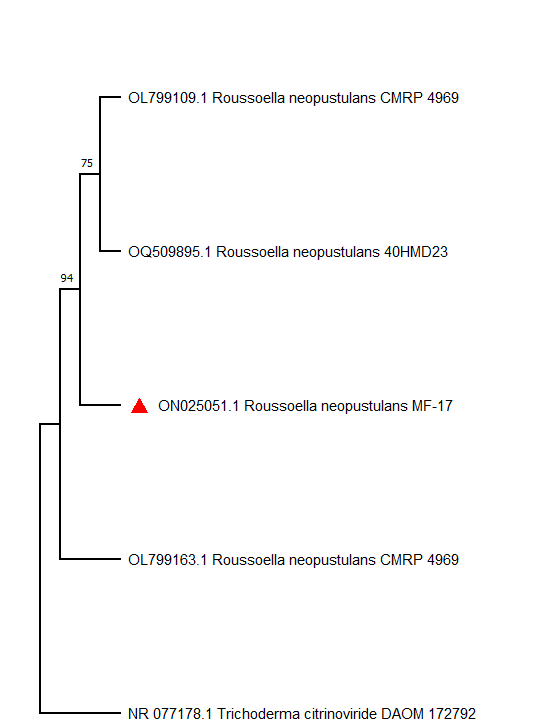**i)** | 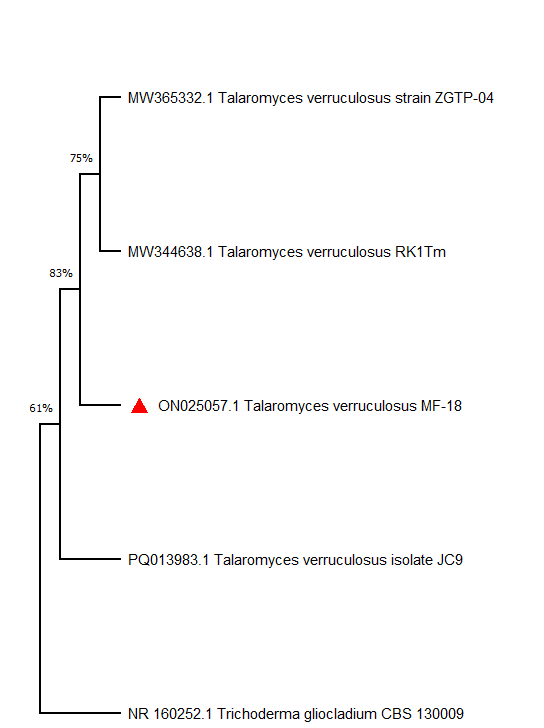**j)** |
| --- | --- |
| 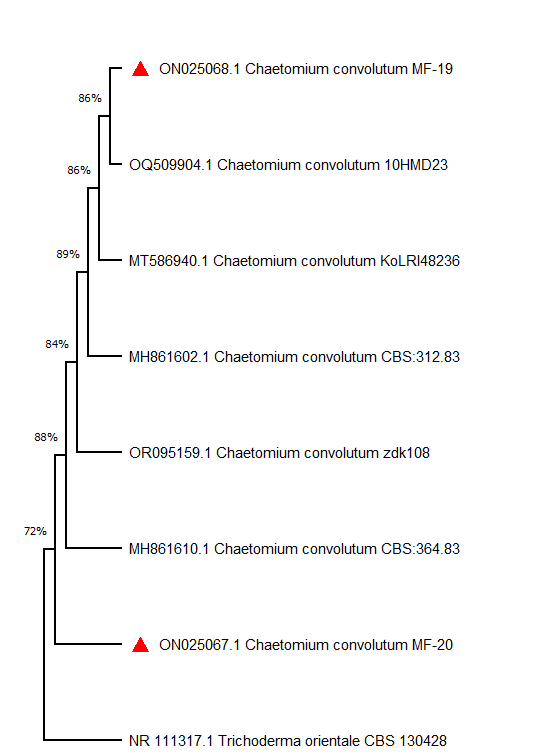 **k)** | |

**SUPPLEMENTARY FIGURE S2 |** Phylogenetic characterization of different mycoparasites. a) *C. minitans*, b)*, P. minitans,* c) *C. rosea,* d) *C. cladosporioides,*e) *P. sclerotiorum,* f) *E. nigrum*, g) *Phoma sp.,* h) *D. glomerata* and *D. rhei,* i) *R. neopustulans,* j) *T. verruculosus*, k) *C. convolutum*

**
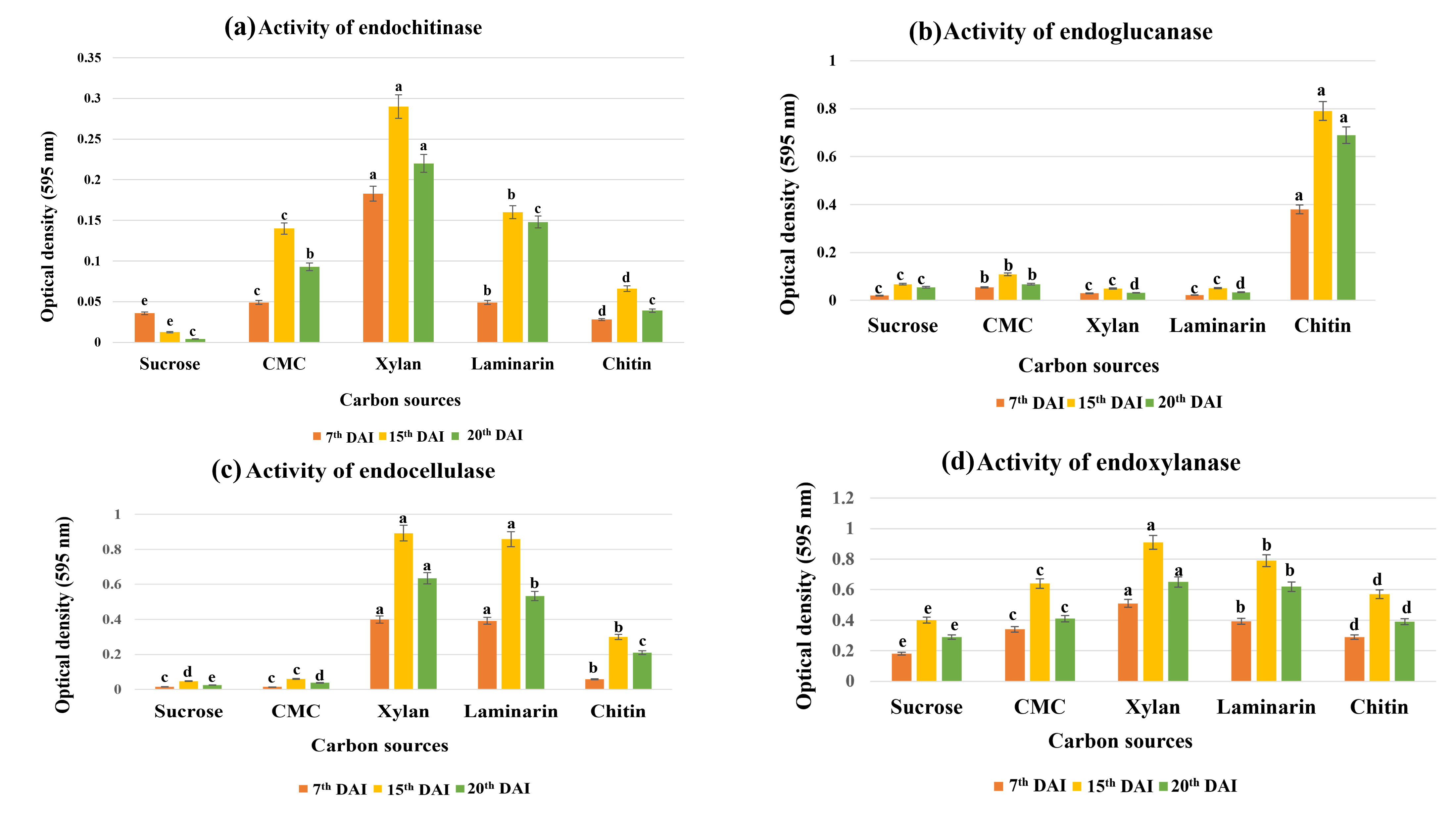
**

**SUPPLEMENTARY FIGURE S3 |** Enzymatic activity of *P. minitans* TNAU-CM 1 grown in PDB supplemented with different carbon sources, recorded at 7^th^, 10^th^, 15^th^, and 20^th^ days after inoculation (DAI). (a) Endochitinase activity, (b) Endoglucanase activity, (c) Endocellulase activity, and (d) Endoxylanase activity. Different carbon sources were used to evaluate their role in inducing specific enzyme systems. Time intervals were selected to capture the dynamics of enzyme production. Values represent the mean ± SE of three replications; Different letters above bars indicate significant differences at p < 0.05.

**
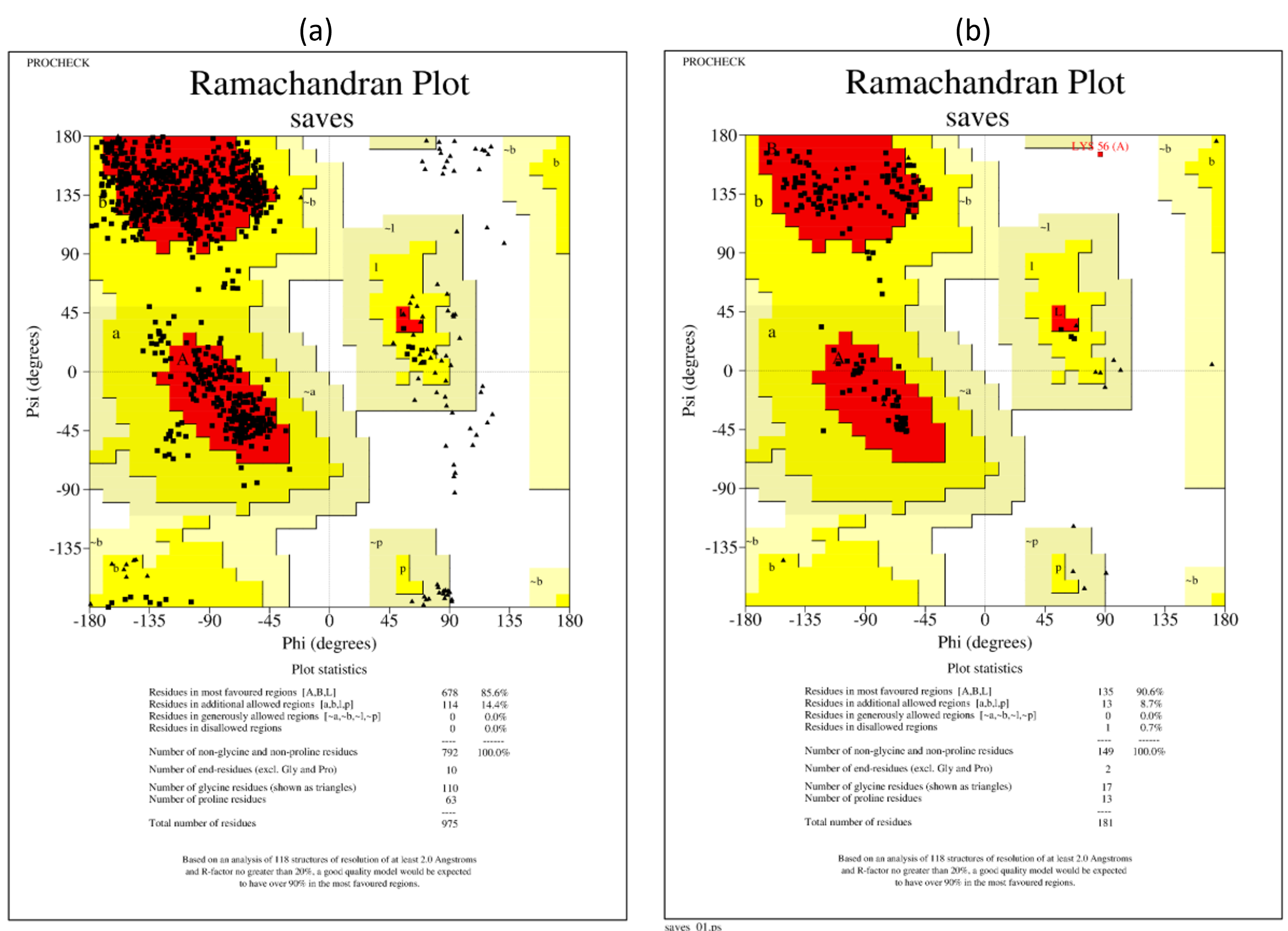
**

**SUPPLEMENTARY FIGURE S4 |** Ramachandran plot validation of protein structures. Ramachandran plots showing stereochemical quality of (a) 1ZNP template protein and (b) modelled SsYCP1. The majority of residues lie in the most favoured regions, with no residues in disallowed regions for SsYCP1, confirming the structural reliability of both models for docking analysis.


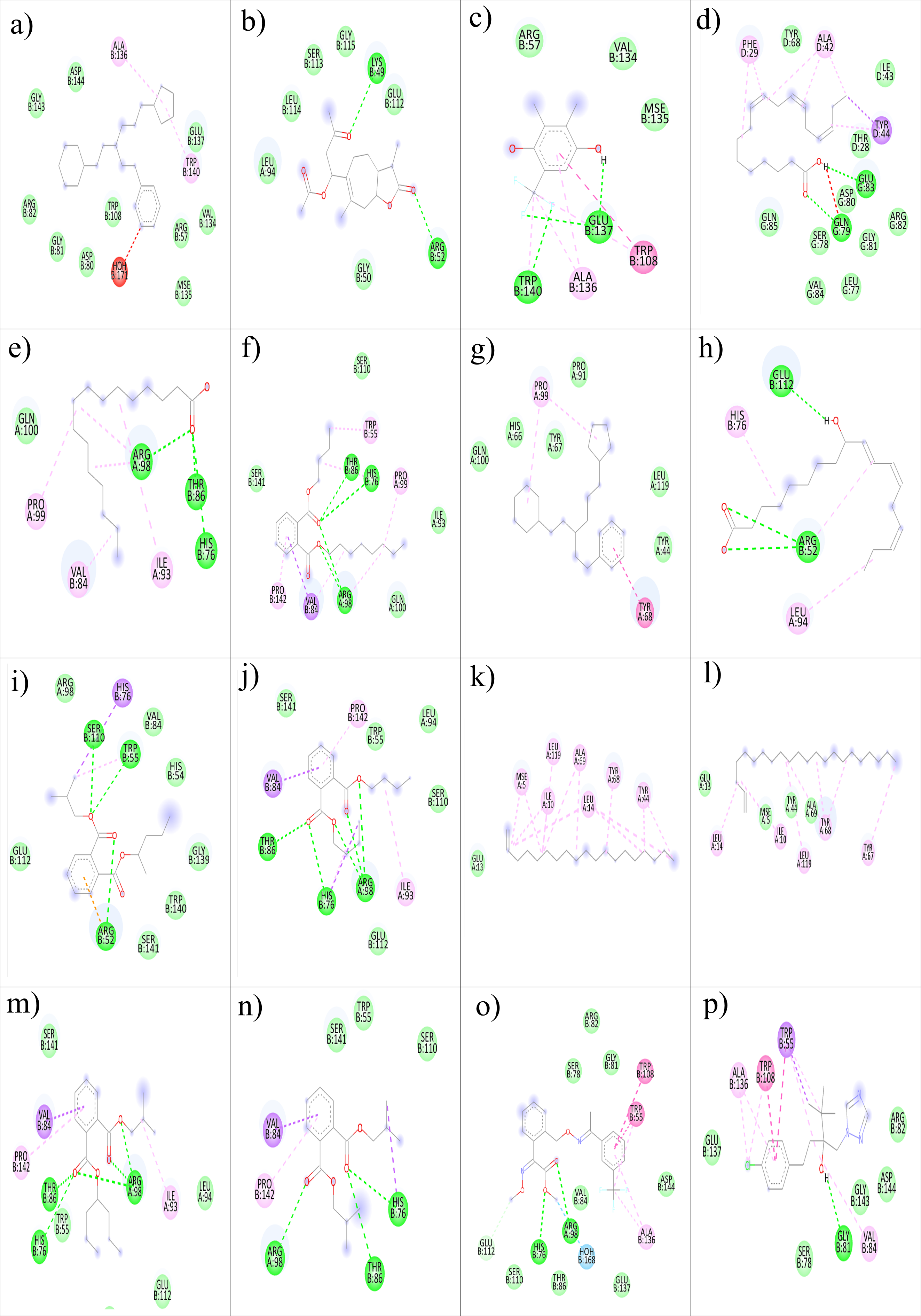


**SUPPLEMENTARY FIGURE S5 |** Molecular docking interactions (2D) of secondary metabolites from TNAU-CM 1 with the 1ZNP protein. (a) Benzene, [3-(2-cyclohexylethyl)-6-cyclopentylhexyl]. (b) Dihydroxanthin. (c) Phen-1,4-diol, 2,3-dimethyl-5-trifluoromethyl. (d) Linolenic Acid. (e) Palmitic Acid. (f) Butyl octyl phthalate. (g) Allyl formate. (h) 1-beta-D-Ribofuranosyl-2,4(1H,3H)-pyrimidinedione. (i) Phthalic acid, isobutyl 2-pentyl ester. (j) Dibutyl Phthalate. (k) Diisobutyl phthalate. (l) 1-Nonadecene. (m) 1-Docosene. (n) Phthalic acid, hept-4-yl isobutyl ester. (o) Trifloxystrobin. (p) Tebuconazole.

| 1. **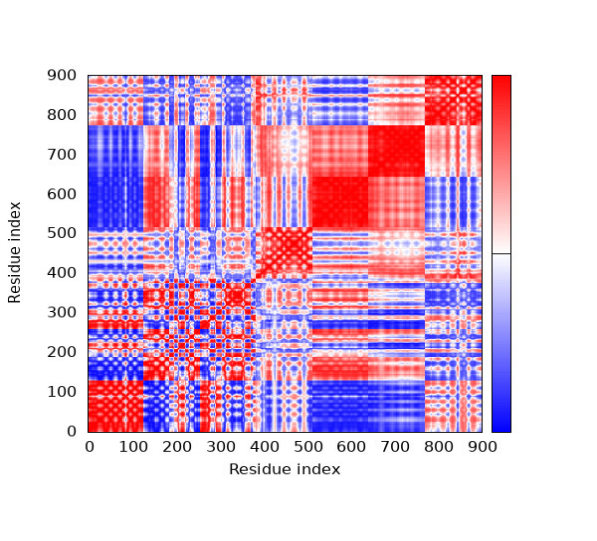** | 1. **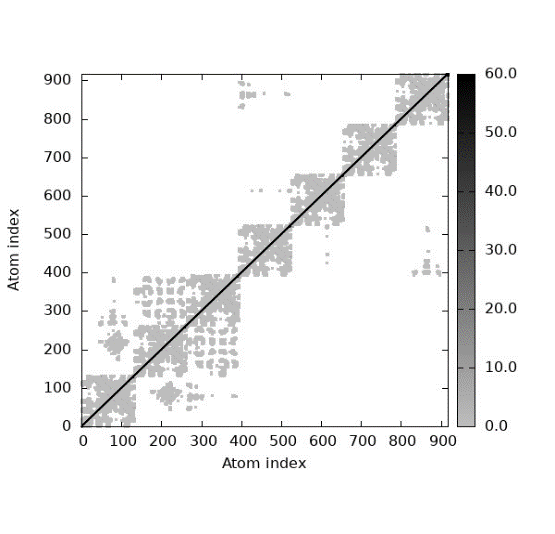** |
| --- | --- |
| 1. **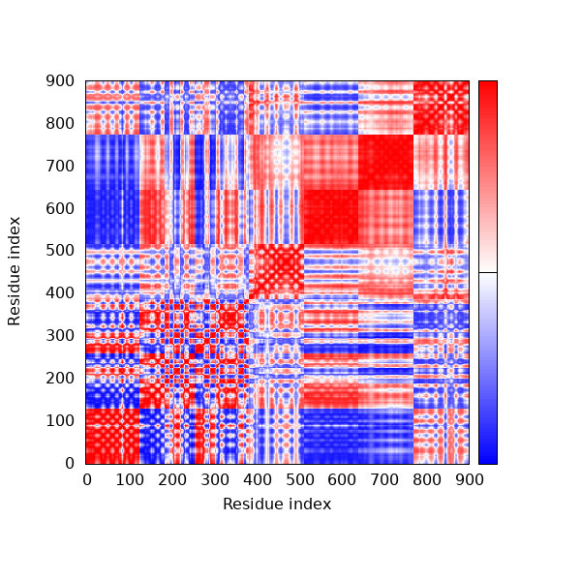** | 1. **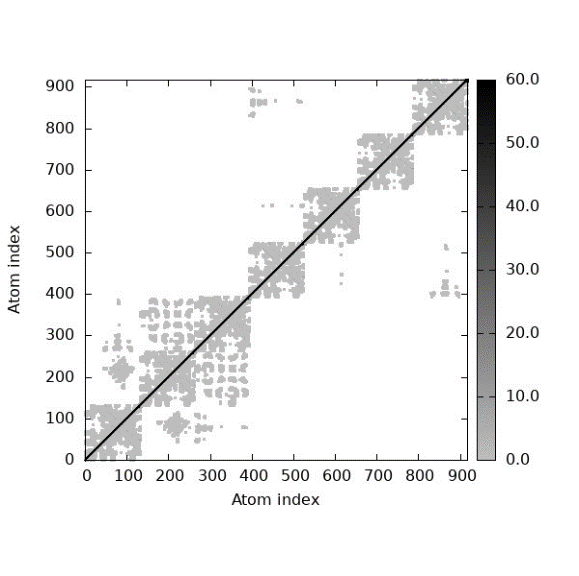** |

**SUPPLEMENTARY FIGURE S6 |** MD simulation of Linoleic acid-1ZNP and Butyl octyl phthalate-1ZNP complexes. (a, b) covariance map and elastic network model of Linoleic acid-1ZNP; (c, d) covariance map and elastic network model of Butyl octyl phthalate-1ZNP.


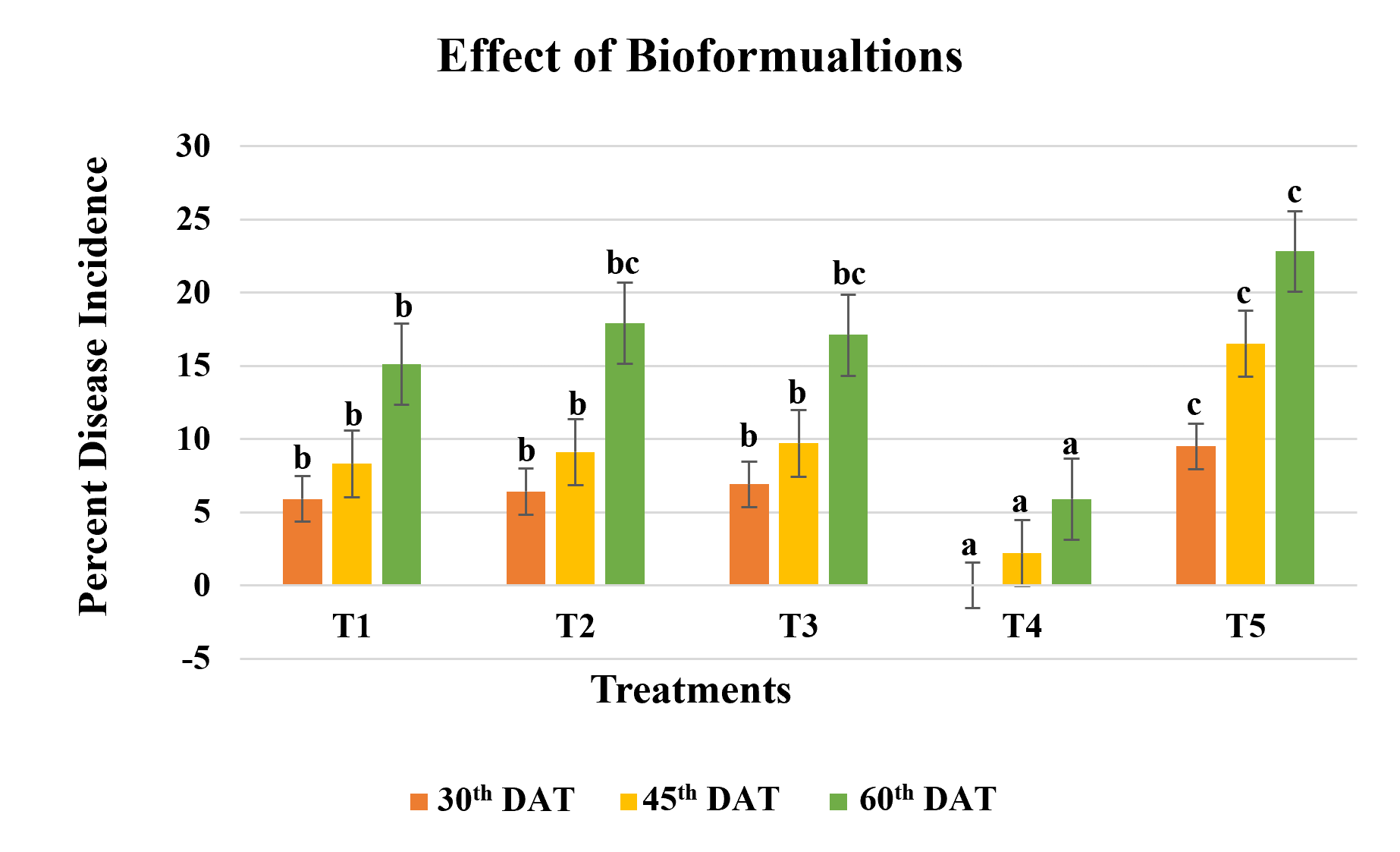


**SUPPLEMENTARY** **FIGURE S7 |** Efficacy of bioformulation against cabbage head rot under greenhouse conditions. T1 - Foliar spray of *P. minitans* (TNAU-CM 1) stock at 5ml/l, T2 - Foliar spray of *T. asperellum* (TRI 15) at 5ml/l, T3 - Foliar spray of *B. subtilis* (Bbv 57) at 5ml/l, T4 - Foliar spray of Tebuconazole + Trifloxystrobin at 1.5 g/l and T5 - Inoculated control without any treatments. Data represent mean ± SE of three replications. Percent disease incidence was subjected to two-way ANOVA (treatment×time), and means were separated using Tukey’s HSD test (p ≤ 0.05). Different letters above bars indicate significant differences among treatments at each time point.


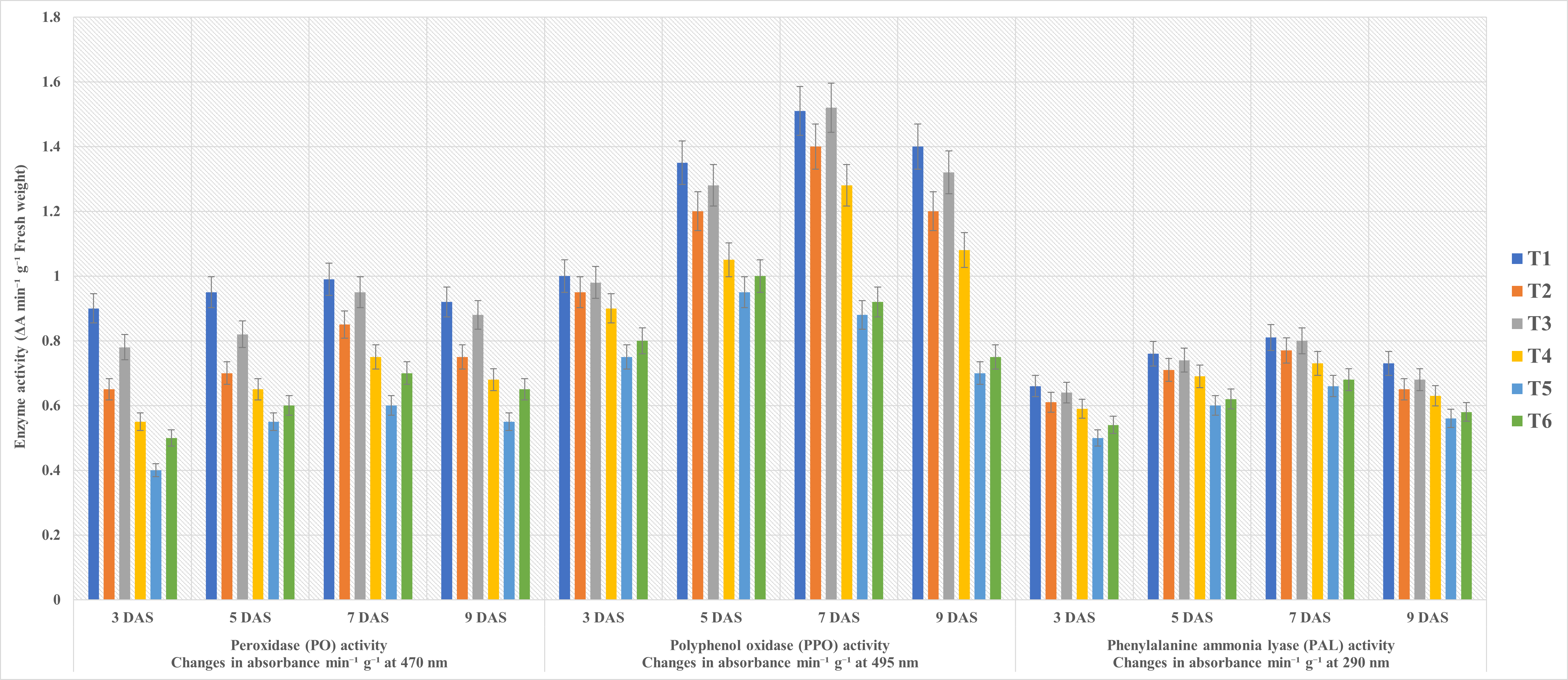


**SUPPLEMENTARY FIGURE S8 | Induction of defense-related enzymes in cabbage treated With *P. minitans* TNAU-CM 1.** T1 – Foliar spray of *P. minitans* (TNAU-CM 1) at 5ml/l, T2 - Foliar spray of *T. asperellum* (TRI 15) at 5ml/l, T3 - Foliar spray of *B. subtilis* (Bbv 57) at 5ml/l, T4 - Foliar spray of Tebuconazole + Trifloxystrobin at 1.5 g/l, T5 - Inoculated control without any treatments, and T6 – Healthy uninoculated control
