# Supplementary material for: Exploiting Paraphaeosphaeria minitans and Its Antifungal Metabolites as Bio‐Fungicides for Eco‐Friendly Management of Head Rot Disease in Cabbage
Source: Microb Biotechnol. 2026 Jan 30;19(2):e70309. doi: 10.1111/1751-7915.70309 (PMC12859388; doi:10.1111/1751-7915.70309)
Supplement: Supplementary file 2 — Table S1: Morphological and molecular characterisation of mycoparasitic fungal isolates. Table S2: Population of P. minitans (TNAU‐CM 1) in different liquid medium on 25th DAI. [file MBT2-19-e70309-s002.doc]

**Supplementary table S1 |** Morphological and molecular characterization of mycoparasitic fungal isolates

| **Sl. no.** | **Isolate** | **Colony morphology** | **Spore / structure** | **Fungal species** | **Accession no.** | **Closest reference (NCBI)** | **Reference accession no.** | **Similarity (%)** |
| --- | --- | --- | --- | --- | --- | --- | --- | --- |
| 1 | TNAU-CM 1 | Ostiolate, globose pycnidia | Abundant conidia | *Paraphaeosphaeria minitans* | OL614782 | Strain P4 | MN795740 | 89 |
| 2 | TNAU-CM 2 | Greyish white colonies with aggregated mycelia | Conidia sparse | *P. minitans*. | OL614980 | Strain P4 | MN795740 | 89 |
| 3 | CM 6 | Whitish, mildly cottony mycelia | Hyaline conidia | *P. minitans* | ON025056 | Strain CB 111750 | MH862883 | 60 |
| 4 | TNAU-CR 01 | Fast-growing pink colonies with branched conidiophores | Elliptical, hyaline conidia | *Clonostachys rosea* | MZ754407 | Isolate 67-1 | KP274075 | 70% |
| 5 | TNAU-CR 02 | Fast-growing pink colonies with branched conidiophores | Elliptical, hyaline conidia | *C. rosea* | ON025052 | Isolate 67-1 | KP274075 | 70 |
| 6 | TNAU-CR 03 | Fast-growing pink colonies with branched conidiophores | Elliptical, hyaline conidia | *C. rosea* | ON025053 | Isolate 67-1 | KP274075 | 70 |
| 7 | TNAU-CR 04 | Fast-growing pink colonies with branched conidiophores | Elliptical, hyaline conidia | *C. rosea* | ON025054 | Isolate 67-1 | KP274075 | 70 |
| 8 | TNAU-CR 05 | Fast-growing pink colonies with branched conidiophores | Elliptical, hyaline conidia | *C. rosea* | ON025055 | Isolate 67-1 | KP274075 | 70 |
| 9 | MF-8 | Greenish colonies | Brush-like conidiophores | *Penicillium sclerotiorum* | ON062087 | Strain E23Y 1A | MW090660 | 52 |
| 10 | MF-9 | Pigmented colonies | Dark multicellular conidia | *Epicoccum nigrum* | ON024790 | Strain F48-04 | KX664419 | 96 |
| 11 | MF-10 | Compact dark colonies | Abundant hyaline conidia | *Phoma herbarum* | ON062193 | Strain YE3135 | MH477285 | 68 |
| 12 | MF-11 | Olive-green, velvety colonies | Chains of lemon-shaped conidia | *Cladosporium cladosporioides* | ON045141 | Strain F47-03 | KX664414 | 95 |
| 13 | MF-12 | Olive-green, velvety colonies | Chains of lemon-shaped conidia | *C. cladosporioides* | ON024886 | Strain F47-03 | KX664414 | 95 |
| 14 | MF-13 | Dark colonies with pycnidia | Ellipsoidal, hyaline conidia | *Phoma sp.* | ON025047 | Strain YE3135 | MH477285 | 68 |
| 15 | MF-14 | Dark colonies with pycnidia | Ellipsoidal, hyaline conidia | *Phoma sp.* | ON025046 | Strain YE3135 | MH477285 | 68 |
| 16 | MF-15 | Crust-like colonies | Dark fruiting bodies, bicellular spores | *Didymella rhei* | ON025049 | Strain PLS3 | OM952211 | 67 |
| 17 | MF-16 | Ash-coloured colonies | Abundant hyaline conidia | *D. glomerata* | ON025050 | Strain PLS3 | OM952211 | 67 |
| 18 | MF-17 | Crust-like colonies | Dark fruiting bodies, bicellular spores | *Roussoella neopustulans* | ON025051 | Strain CMRP 4969 | OL799163 | 75 |
| 19 | MF-18 | Greenish colonies | Brush-like conidiophores | *Talaromyces verruculosus* | ON025057 | Strain RK1Tm | MW344638 | 83 |
| 20 | MF-19 | Spreading growth with perithecia, spiral appendages | Lemon-shaped spores | *Chaetomium convolutum* | ON025068 | Isolate 10HMD23 | OQ509904 | 86 |
| 21 | MF-20 | Spreading growth with perithecia, spiral appendages | Lemon-shaped spores | *C. convolutum* | ON025067 | Isolate CBS 364.83 | MH861610 | 72 |

**Supplementary table S2 |** Population of *P. minitnas* (TNAU-CM 1) in different liquid medium on 25^th^ DAI

| **Sl. no.** | **Liquid medium** | ***P. minitans* (TNAU-CM 1)** | | |
| --- | --- | --- | --- | --- |
|  |  | **Dry mycelial weight (g/l)*** | **Sporulation (10^8^ spores/ml)*** | |
| 1 | Malt yeast broth | 11.14^d^  (3.33) | | 7.26^d^  (2.78) |
| 2 | Nutrient yeast sucrose broth | 12.96^c^  (3.60) | | 8.11^c^  (2.93) |
| 3 | Molasses yeast broth | 16.41^a^  (4.05) | | 13.21^a^  (3.70) |
| 4 | Jaggery yeast broth | 13.73^b^  (3.70) | | 10.26^b^  (3.28) |
| CD (P≤ 0.05) | | 0.029 | | 0.078 |
| SED | | 0.000 | | 0.027 |

*Values are means of four replications

Figures in the parentheses are square root transformed values

Means in a column followed by same superscript alphabet are not significantly different as per DMRT at P≤ 0.05
